# Molecular Dialogues between Early Divergent Fungi and Bacteria in an Antagonism versus a Mutualism

**DOI:** 10.1128/mBio.02088-20

**Published:** 2020-09-08

**Authors:** Olga A. Lastovetsky, Lev D. Krasnovsky, Xiaotian Qin, Maria L. Gaspar, Andrii P. Gryganskyi, Marcel Huntemann, Alicia Clum, Manoj Pillay, Krishnaveni Palaniappan, Neha Varghese, Natalia Mikhailova, Dimitrios Stamatis, T. B. K. Reddy, Chris Daum, Nicole Shapiro, Natalia Ivanova, Nikos Kyrpides, Tanja Woyke, Teresa E. Pawlowska

**Affiliations:** a Graduate Field of Microbiology, Cornell University, Ithaca, New York, USA; b School of Integrative Plant Science, Plant Pathology & Plant-Microbe Biology, Cornell University, Ithaca, New York, USA; c Department of Biology, Duke University, Durham, North Carolina, USA; d U.S. Department of Energy Joint Genome Institute, Berkeley, California, USA; University of California, Berkeley

**Keywords:** cell wall remodeling, innate immunity, *Mycetohabitans*, reactive oxygen species, *Rhizopus microsporus*, cell wall remodeling

## Abstract

Fungal-bacterial symbioses range from antagonisms to mutualisms and remain one of the least understood interdomain interactions despite their ubiquity as well as ecological and medical importance. To build a predictive conceptual framework for understanding interactions between fungi and bacteria in different types of symbioses, we surveyed fungal and bacterial transcriptional responses in the mutualism between Rhizopus microsporus (*Rm*) (ATCC 52813, host) and its *Mycetohabitans* (formerly *Burkholderia*) endobacteria versus the antagonism between a nonhost *Rm* (ATCC 11559) and *Mycetohabitans* isolated from the host, at two time points, before and after partner physical contact. We found that bacteria and fungi sensed each other before contact and altered gene expression patterns accordingly. *Mycetohabitans* did not discriminate between the host and nonhost and engaged a common set of genes encoding known as well as novel symbiosis factors. In contrast, responses of the host versus nonhost to endobacteria were dramatically different, converging on the altered expression of genes involved in cell wall biosynthesis and reactive oxygen species (ROS) metabolism. On the basis of the observed patterns, we formulated a set of hypotheses describing fungal-bacterial interactions and tested some of them. By conducting ROS measurements, we confirmed that nonhost fungi increased production of ROS in response to endobacteria, whereas host fungi quenched their ROS output, suggesting that ROS metabolism contributes to the nonhost resistance to bacterial infection and the host ability to form a mutualism. Overall, our study offers a testable framework of predictions describing interactions of early divergent Mucoromycotina fungi with bacteria.

## Abstract (precis)

Animals and plants interact with microbes by engaging specific surveillance systems, regulatory networks, and response modules that allow for accommodation of mutualists and defense against antagonists. Antimicrobial defense responses are mediated in both animals and plants by innate immunity systems that owe their functional similarities to convergent evolution. Like animals and plants, fungi interact with bacteria. However, the principles governing these relations are only now being discovered. In a study system of host and nonhost fungi interacting with a bacterium isolated from the host, we found that bacteria used a common gene repertoire to engage both partners. In contrast, fungal responses to bacteria differed dramatically between the host and nonhost. These findings suggest that as in animals and plants, the genetic makeup of the fungus determines whether bacterial partners are perceived as mutualists or antagonists and what specific regulatory networks and response modules are initiated during each encounter.

## INTRODUCTION

Reciprocal communication between interacting partners is a feature central to all interspecific symbioses, antagonisms and mutualisms alike (1–5). As partners approach each other and come into contact, they exchange biochemical signals repeatedly throughout the duration of the relationship. Many molecular conversations guiding interactions of prokaryotes with animals and plants have been deciphered, revealing that both mutualisms (1, 4) and antagonisms (3, 5) are initiated by the host perception of microbe-associated molecular patterns (MAMPs) and effectors. In mutualisms, symbiont accommodation leads to exchange of goods and services between the partners (1, 4). In antagonisms, biotrophic exploitation of the host resources is possible only for microbial invaders that are able to overcome host defenses preventing unwanted microbes from entering host cells (6, 7) and eliminating attackers through programmed death of the infected cells (3, 5). Remarkably, despite functional similarities in surveillance systems, response regulatory networks and defense modules, innate immunity systems of animals and plants are products of convergent evolution (8–10).

Like animals and plants, fungi engage in interactions with bacteria. However, in contrast to animals and plants, fungal-bacterial symbioses remain poorly understood (11, 12). While no general principles governing these interactions have been identified thus far, several shared patterns have emerged. For example, fungi are able to perceive secreted secondary metabolite signals (13–16) and MAMPs (17, 18) of bacterial origin and respond to them with alterations of metabolism (14, 18), morphology (15–17), and growth kinetics (16). Interestingly, some of the reactions induced by bacterial antagonists appear to resemble innate immunity responses of animals and plants, including mitochondrial membrane polarization (18) as well as activation of genes responsible for sequestration of iron (18) and biosynthesis of antibacterial secondary metabolites (18, 19). In addition, fungi are capable of detoxifying antifungal secondary metabolites produced by interacting bacteria (18–20).

Among fungal-bacterial interactions, the mutualism between Rhizopus microsporus (*Rm*) (Mucoromycotina) and *Mycetohabitans* sp. endobacteria (*Betaproteobacteria*; previously classified as *Burkholderia*) is emerging as a model for the study of fungal-bacterial relationships (12, 21–25). In this symbiosis, the fungal host can be cured of its bacterial symbionts, the endobacteria extracted and cultivated independently, and the symbiosis reassembled with ease (21). The molecular bases governing the establishment of the *Rm-Mycetohabitans* symbiosis are beginning to be unraveled, with much progress being made in understanding the bacterial factors necessary to invade the fungus (23, 26, 27) and the fungal changes that occur to accommodate endobacteria (24). In particular, we previously showed that the high-osmolarity glycerol (HOG) mitogen-activated protein kinase (MAPK) signaling pathway and specific changes in lipid metabolism of the host are important for the mutualism establishment (24). We were able to make these observations because, in addition to *Rm* host isolates that naturally harbor endobacteria, there exist closely related but naturally bacterium-free nonhost isolates that interact antagonistically with endobacteria of the host and do not become infected by them (24). Importantly, other Mucoromycotina fungi display a similar antagonistic reaction toward endobacteria of *Rm* (24).

To start building a predictive framework for understanding fungal-bacterial interactions, we conducted global profiling of fungal and bacterial transcriptional responses in the antagonistic versus mutualistic interaction of closely related *Rm* isolates with *Mycetohabitans* at two time points, when partners interact at a distance before they come to physical contact (precontact) and upon physical contact. We dissected molecular dialogues between the partners and formulated a set of hypotheses regarding bacterial and fungal factors contributing to antagonism versus mutualism. As well as unraveling the molecular similarities between these two interactions, we aimed to understand how the nonhost is able to resist infection by bacteria that are mutualists of the host. We found that in interactions with the nonhost and host, the symbiotic bacteria engaged a common set of genes encoding known as well as novel symbiosis factors. In contrast, nonhost and host responses, albeit almost entirely different at the gene level, converged on the altered expression of genes involved in cell wall biosynthesis and reactive oxygen species (ROS) metabolism. Specifically, the nonhost upregulated the expression of genes involved in ROS production, while the host upregulated ROS detoxification. On the basis of these observations, we formulated the hypotheses that the nonhost responds to *Mycetohabitans* by increasing the ROS output, whereas the host quenches ROS. Our empirical experiments revealed that, indeed, the nonhost increased its ROS output during interaction with bacteria, which may contribute to its ability to resist bacterial infection. In contrast, the host fungus responded to bacteria with a reduced ROS output and was able to form a mutualism. Collectively, our observations offer a framework of testable predictions describing interactions of early divergent Mucoromycotina fungi with bacteria. They also suggest that fungal responses may involve mechanisms that resemble innate immunity mechanisms of plants and animals.

## RESULTS AND DISCUSSION

**FIG 1:**
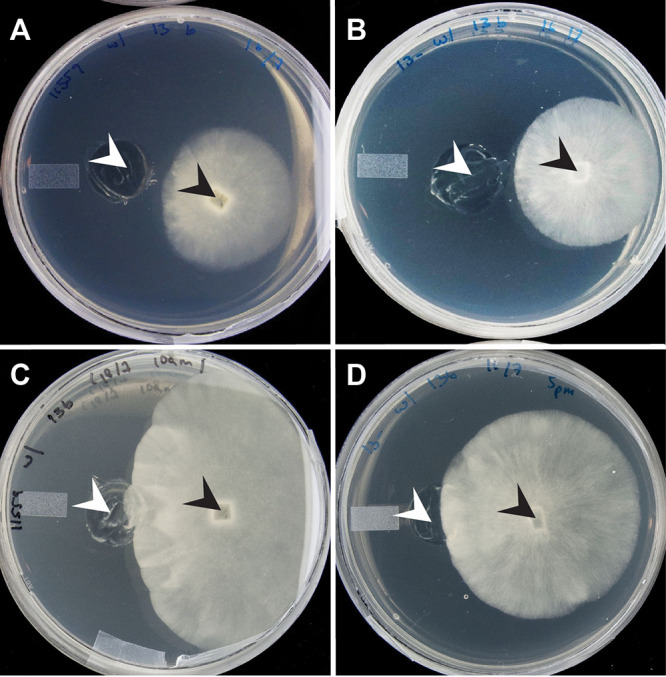
Positions of the nonhost *Rm* ATCC 11559 and the previously cured host *Rm* ATCC 52813 relative to *Mycetohabitans* sp. B13 endobacteria isolated from the host ATCC 52813 at the time of harvest for the RNA-seq experiment before contact and after physical contact. (A) The nonhost interacting with *Mycetohabitans* precontact. (B) The host interacting with *Mycetohabitnas* precontact. (C) The nonhost interacting with *Mycetohabitans* after contact. (D) The host interacting with *Mycetohabitans* after contact. White arrowheads indicate sites of bacterial inoculation, and black arrowheads point to sites of fungal inoculation.

To identify fungal and bacterial genes involved in the antagonistic and mutualistic interactions of the *Rm* nonhost (ATCC 11559) and previously cured *Rm* host (ATCC 52813) with *Mycetohabitans* sp. strain B13 endobacteria isolated from the host, we analyzed expression changes during partner cocultivation at two time points: when the partners interacted at a distance (precontact) and after physical contact (Fig. 1). Fungal transcriptome sequencing (RNA-seq) reads were mapped to genomes of *Rm* ATCC 11559 (NCBI accession number PRJNA330885) and *Rm* ATCC 52813 (NCBI accession PRJNA205957). *Mycetohabitans* endobacterial reads were mapped to the genome of *Mycetohabitans* sp. B13 (endobacteria of the host *Rm* ATCC 52813, NCBI accession number PRJNA303198, see Table S1 in the supplemental material). It should be noted that *Mycetohabitans* sp. B13 likely represents a novel species, as the whole-genome average nucleotide identity (ANI) values that it shares with closely related Mycetohabitans rhizoxinica HKI 454 (28, 29) and Mycetohabitans endofungorum HKI 456 (28, 30) are 91% and 94.6%, respectively. These values do not exceed the 95 to 96% similarity threshold accepted as a counterpart of a DNA-DNA hybridization value of 70% for species delineation (31). Differentially expressed (DE) fungal genes were identified by comparing expression profiles of host and nonhost fungi grown alone to host and nonhost fungi cocultivated with endobacteria. Similarly, DE endobacterial genes were identified by comparing endobacteria grown alone to endobacteria cocultivated with either host or nonhost fungi. A false discovery rate of 0.01 was used as a cutoff for identification of DE genes.

10.1128/mBio.02088-20.2 TABLE S1 Genome statistics for *Mycetohabitans* sp. B13 and *Mycetohabitans* sp. B14 as well as previously sequenced *Mycetohabitans* spp. included for comparison. Download Table S1, PDF file, 0.04 MB. Copyright © 2020 Lastovetsky et al. 2020 Lastovetsky et al. This content is distributed under the terms of the Creative Commons Attribution 4.0 International license.

### Precontact transcriptional responses in fungi.

The antagonistic interaction between the nonhost and endobacteria of the host was marked by changes in the fungal colony morphology, whereby the colony developed a zone of reduced growth (24). This pattern was more pronounced when the fungus neared the bacteria (24), but it was also visible before partners came into physical contact (Fig. 1). No such growth alterations occur in the host during interaction with its endobacteria. These phenotypic patterns were consistent with the magnitude of transcriptomic responses of 145 DE genes in the nonhost (122 upregulated) and 10 DE genes in the host (6 upregulated), suggesting, in turn, that both nonhost and host fungi were able to sense bacterial presence at a distance.

**(i) Detoxification genes.** The precontact appearance of growth alterations in the nonhost colonies is unlikely to be the result of local nutrient or water depletion by the bacteria, in which case a similar growth alteration response would also be seen in the host. Instead, analysis of the DE genes by the nonhost suggested a reaction to a bacterial metabolite, supported by the upregulated expression of various genes encoding transporters (Protein identifiers [IDs] 224825, 205631, 254867, 170380, 245627) and a detoxification enzyme, glutathione *S*-transferase (GST) (216648). Overexpression of transporters is a well-known feature of drug resistance in fungi (32), whereas GST genes are typically overexpressed during chemical challenge and oxidative stress response (33, 34). We did not detect differential expression of any secondary metabolism gene clusters encoded in the endosymbiont genome, suggesting that the nonhost responded to a constitutively produced bacterial compound, which remains unknown.

**(ii) Cell wall biogenesis genes.** While both nonhost and host upregulated genes involved in cell wall synthesis and remodeling, there was no overlap between these gene sets (Table 1). Fungal cell walls are generally composed of a scaffold of cross-linked polysaccharides, such as glucans, chitin, chitosan, and a matrix of proteins and mannans (35, 36). In the nonhost, we detected differential expression of genes with chitin-modifying function, such as chitinases, which break down chitin, and chitin deacetylases, which produce a deacetylated form of chitin known as chitosan, a structural component of *Rhizopus* cell walls (37) (Table 1). Importantly, in many fungi, chitinases are tightly spatiotemporally controlled at the transcription level (38). Moreover, chitin deacetylase transcription levels correlate with activity as well as chitosan content of fungal cell walls (39). While expression of chitinases was both up- and downregulated, chitin deacetylases were mostly downregulated. In addition to altered expression of genes encoding chitin-modifying enzymes, the nonhost upregulated seven genes with mannosyltransferase activity and four septin genes, all involved in cell wall remodeling, strengthening, and hyphal growth (35, 40). Even though the host precontact response was more limited, some of those genes also had cell wall-related function (Table 1). Specifically, in contrast to the nonhost, the host upregulated genes encoding chitin synthase and chitin deacetylase, suggesting synthesis of both chitin and chitosan. Overall, the nonhost and host precontact responses to endobacteria converged on cell wall remodeling, albeit of different types, highlighting the difference between these two types of interactions. Some of the nonhost responses were most likely related to the colony growth alterations, while others may reflect changes in the cell wall composition itself. We speculate that perception of an antagonistic bacterium prompted the nonhost to alter its cell wall for protection from bacterial invasion, whereas perception of a mutualistic partner induced the host to initiate cell wall changes to facilitate symbiont entry.

**TABLE 1:** Cell wall-related genes DE in the host (ATCC 52813) and the nonhost (ATCC 11559) during precontact interaction with *Mycetohabitans* sp. B13

Protein ID	Log_2_ FC^*a*^	FDR^*b*^	CAZY/ InterPro/ PFAM	Annotation^*c*^
Host				
241339	1.41	4.05E−02	GT2	Chitin synthase*
245394	0.99	7.20E−03	CE4	Chitin deacetylase**
252367	0.67	3.89E−02	PF10342	Mixed-link glucanase
Nonhost				
178226	1.84	1.85E−04	GT15	2-Alpha-mannosyltransferase*
177931	1.75	3.20E−05	GT15	2-Alpha-mannosyltransferase*
177938	1.67	1.42E−04	GT15	2-Alpha-mannosyltransferase*
290291	1.62	2.15E−04	GT15	2-Alpha-mannosyltransferase*
210844	1.48	4.85E−05	GH16	Xylanase/beta(1,2-1,4)glucanase**
169859	1.47	4.95E−04	GT39	Mannosyltransferase activity*
118361	1.45	4.84E−04	GT39	Mannosyltransferase activity*
71975	1.37	3.58E−04	GH47	Alpha-1,2-mannosidase**
177895	1.27	2.15E−04	IPR016491	Septin
241953	1.25	1.58E−03	GT15	2-Alpha-mannosyltransferase*
195250	1.20	4.63E−03	IPR016491	Septin
5395	1.17	2.15E−04	IPR016491	Septin
198617	1.08	2.57E−03	IPR016491	Septin
113619	1.07	9.37E−03	GH18	Chitinase**
288806	1.01	7.25E−03	CE4	Chitin deacetylase**
178335	−0.82	6.15E−03	CE4	Chitin deacetylase**
129845	−0.85	3.96E−03	GH18	Chitinase**
203498	−0.87	7.20E−03	CE4	Chitin deacetylase**
50892	−0.97	1.27E−03	CE4	Chitin deacetylase**
209253	−1.58	3.94E−07	CE4	Chitin deacetylase**
97566	−2.12	1.02E−03	CE4	Chitin deacetylase**
204982	−2.24	8.05E−06	GH28	Endo-polygalacturonase**

a Log_2_ FC, log_2_ fold change. Positive log_2_ FC values denote upregulated genes, and negative log_2_ FC values denote downregulated genes.

b FDR, false discovery rate.

c Annotations obtained by using CAZYmes Analysis Toolkit (109) are indicated by an asterisk. Annotations obtained through the *myco*CLAP database (110) are indicated by two asterisks. The remaining annotations are from JGI.

### Physical contact transcriptional responses in fungi.

In contrast to the precontact interaction with endobacteria, during physical contact, the nonhost DE less than half of the number of genes as the host. In our previous work, we analyzed the fungal responses to bacteria and showed that different sets of genes were engaged in host and nonhost fungi during physical contact (24). In particular, our previous work identified the activation of genes in the HOG MAPK signaling pathway and specific changes in lipid metabolism in the host (24). In the present study, we analyzed the remaining genes that were DE due to bacteria in the host and nonhost fungi and were not part of the lipid metabolism or HOG MAPK signaling pathway.

**(i) Genes involved in cyclic AMP signaling.** In addition to the HOG MAPK signaling cascade, genes encoding other signaling cascades were DE during the mutualistic interaction with endobacteria (Table S2). Cyclic AMP (cAMP) signaling, for example, likely played a role. cAMP is a secondary messenger synthesized by adenylyl cyclases in response to activation by heterotrimeric G proteins (41) or small GTPases of the Ras superfamily (42). cAMP directly activates cAMP-dependent kinases, which, in turn, phosphorylate their protein targets critical for fungal growth and development (41). We detected upregulation of genes encoding two cAMP-dependent protein kinases (286008 and 278678) and two small G proteins of the Ras superfamily (136105 and 250648) as well as many small G protein regulators, such as guanine nucleotide exchange factors, GEFs, which activate G protein signaling, and GTPase-activating proteins, GAPs, that downregulate G protein signaling (Table S2). Many other signaling genes, largely encoding protein kinases, were also DE during the mutualistic interaction. Given an expanded repertoire and poor characterization of these signaling genes in Mucoromycotina (43), it is hard to speculate as to their function. Moreover, signaling cascades and activity of protein kinases are regulated at multiple levels in eukaryotic cells, including phosphorylation, cellular localization, translation, and transcription (44). Consequently, increased levels of transcription of these genes may not be a direct reflection of their activity. However, they provide a basis for formulating hypotheses about the mechanisms underlying the mutualistic response to bacteria.

10.1128/mBio.02088-20.3 TABLE S2 Signaling genes DE in the host *Rm* ATCC 52813 during physical interaction with endobacteria. Download Table S2, PDF file, 0.1 MB. Copyright © 2020 Lastovetsky et al. 2020 Lastovetsky et al. This content is distributed under the terms of the Creative Commons Attribution 4.0 International license.

**(ii) Genes involved in cytoskeletal rearrangements.** In the host, there were many DE genes involved in cytoskeletal rearrangements, the majority of which were upregulated (Table 2). Several interpretations of this pattern are possible. For example, cytoskeletal rearrangements could be linked to cell cycle progression (45). In support of this interpretation, we identified several DE genes involved in the cell cycle, including the cell division gene *cdc15* (232829) (Table S3). This pattern suggests an intriguing possibility that endobacteria interact with the fungal host cell cycle. Alternatively, cytoskeletal rearrangements could be a direct response to endosymbionts, which move in the host cytoplasm even though their genomes do not encode motility features, such as flagella (46). For example, other *Burkholderia* species, such as B. pseudomallei and B. mallei, polymerize host actin for motility (47). However, the endobacteria of *Rm* do not encode the *Burkholderia* intracellular motility A protein BimA requisite for such actin-based mobility. Instead, it is more likely that *Rm* rearranges its cytoskeleton to accommodate endobacteria, a strategy consistent with the lack of differential expression of cytoskeleton-related genes in the nonhost.

**TABLE 2:** Cytoskeleton-related genes DE in the host (ATCC 52813) during physical interaction with *Mycetohabitans* sp. B13

Protein ID	Log_2 _ FC^*a*^	FDR^*b*^	Annotation^*c*^	Organism
252161	2.65	1.07E−07	Rho-GTPase-activating protein BAG7	Saccharomyces cerevisiae
60967	2.56	6.19E−04	Rho1 guanine nucleotide exchange factor 2	Schizosaccharomyces pombe
242325	1.89	1.23E−11	Hypothetical LIM domain-containing protein	Rhizopus microsporus
310169	1.73	5.14E−06	Hypothetical LIM domain-containing protein	Rhizopus microsporus
225711	1.58	1.29E−06	Actinin-like protein	Laccaria bicolor
280334	1.43	1.10E−08	CAP-Gly domain-containing protein	Rhizopus delemar
198343	1.42	3.20E−06	Myosin	Rhizopus microsporus
240217	1.32	9.42E−14	Regulator of cytoskeleton and endocytosis RVS167	Candida albicans
274088	1.21	3.24E−05	Probable Rho-type GTPase-activating protein 2	Schizosaccharomyces pombe
181905	1.20	8.24E−05	Myosin	Rhizopus microsporus
266182	1.19	5.69E−04	Kinase with actin-binding calponin homology domain	*Rhizopus delemar*
128872	1.14	4.00E−03	Hypothetical LIM domain-containing protein	Mucor circinelloides
226218	1.01	4.85E−03	Probable Rho-GTPase-activating protein 7	Schizosaccharomyces pombe
235547	1.00	4.26E−07	BZZ1	Saccharomyces cerevisiae
245977	0.92	4.55E−09	Fimbrin	*Rhizopus delemar*
236201	0.70	1.57E−04	Protein dip1	Schizosaccharomyces pombe
237596	0.69	4.25E−04	Rho-GTPase-activating protein BAG7	Saccharomyces cerevisiae
249809	0.67	1.42E−04	Cofilin/tropomyosin-type actin-binding domain- containing protein	Rhizopus microsporus
291147	0.64	6.85E−03	PH domain-containing protein	Rhizopus microsporus
244149	0.55	8.39E−04	Myosin I	Mucor circinelloides
233557	−0.54	1.36E−03	Tubulin-folding cofactor D	Schizosaccharomyces pombe
236630	−0.63	6.23E−03	Rho guanine nucleotide exchange factor SCD1	Schizosaccharomyces pombe
15091	−0.81	3.17E−03	RhoGAP domain-containing protein	Rhizopus microsporus

a Log_2_ FC, log_2_ fold change. Positive log_2_ FC values denote upregulated genes, and negative log_2_ FC values denote downregulated genes.

b FDR, false discovery rate.

c Annotations were obtained manually by PSI-BLAST searches (102) of the Swiss-Prot/UniProtKB database.

10.1128/mBio.02088-20.4 TABLE S3 Cell cycle-related genes DE in the host (ATCC 52813) during physical interaction with *Mycetohabitans* sp. B13. Download Table S3, PDF file, 0.04 MB. Copyright © 2020 Lastovetsky et al. 2020 Lastovetsky et al. This content is distributed under the terms of the Creative Commons Attribution 4.0 International license.

**(iii) Cell wall biogenesis genes.** As during the precontact interaction (Table 1), cell wall-related genes were involved in the physical contact and enriched among the upregulated fraction in the nonhost (see Fig. S1 in the supplemental material; Table 3). Twenty-six genes with cell wall-related function were DE in the nonhost, and eight genes were DE in the host (Table 3). There was a single chitin synthase gene that was commonly DE in both fungi. Only in the host, we detected overexpression of another chitin synthase gene as well as two genes encoding activators of chitin synthesis, suggesting that the mutualistic interaction with endosymbionts was accompanied by the synthesis of chitin. In other fungi, expression of chitin synthases is known to become upregulated upon cell wall stress and correlates with increased chitin composition of cell walls upon challenge with 1,3-beta-glucan synthase inhibitors (48). In the nonhost, on the other hand, we detected the overexpression of a number of chitinase genes as well as chitin deacetylase genes. Their expression patterns in the nonhost suggest that chitin was broken down and chitosan was made. Additionally, the nonhost appeared to initiate synthesis of 1,3-beta-glucan, evident by the upregulation of 1,3-beta-glucan synthase genes, expression of which also correlates with their activity in other fungi (49). We hypothesize that these different cell wall modifications are related to the different reactions of the two fungi to chitinase, a cell wall-degrading enzyme that M. rhizoxinica uses to gain entry into host hyphae (23). The activation of chitin synthesis by the host could reflect the need to resynthesize the chitin that endobacteria broke down during hyphal entry. It is also possible that by increasing the ratio of chitin relative to other cell wall polysaccharides, the host might facilitate bacterial entry and symbiosis establishment. On the other hand, whereas the nonhost did not become infected by endobacteria, it likely still experienced cell wall stress from the bacterial chitinase, expression of which endobacteria upregulated during physical contact with both fungi. The nonhost response of increasing the ratio of chitosan and 1,3-beta-glucan relative to chitin could thus be a defense strategy, as it would ensure enhanced cell wall stability in the presence of external chitinases. An analogous strategy of altering cell wall ratios of 1,3-beta-glucan to chitin is employed by filamentous fungi in response to cell wall antagonistic drugs, such as inhibitors of chitin synthase or glucan synthase (50).

**TABLE 3:** Cell wall-related genes DE in the host (ATCC 52813) and the nonhost (ATCC 11559) during physical interaction with *Mycetohabitans* sp. B13

Protein ID	Log_2_ FC^*a*^	FDR^*b*^	CAZY/ InterPro	Annotation^*c*^
Host				
308710	0.95	9.18E−03	CE4	Chitin deacetylase**
285170	0.69	8.08E−04	GT15	Alpha-1,2-mannosyltransferase*
202842	1.45	1.77E−07	GT2	Chitin synthase*
211861	0.50	4.47E−03	GH9	Endoglucanase**
284826	1.05	3.90E−05	IPR006597	Extracellular protein SEL-1, homolog of SKT5, activator of chitin synthase in *Saccharomyces* * cerevisiae*
237437	1.02	8.69E−04	IPR006597	Extracellular protein SEL-1, homolog of SKT5, activator of chitin synthase in *Saccharomyces* * cerevisiae*
226625	1.76	6.06E−08	GT2	Chitin synthase
202463	−0.92	1.73E−03	GH15|CBM21	Glucoamylase**
Nonhost				
177895	0.94	5.55E−04	IPR016491	Septin
195250	1.28	4.10E−03	IPR016491	Septin
54483	1.35	1.95E−03	GT48	1,3-Beta-glucan synthase*
224455	1.31	2.10E−04	GT48	1,3-Beta-glucan synthase*
178226	1.27	1.83E−03	GT15	Alpha-1,2-mannosyltransferase*
177931	1.12	3.65E−03	GT15	Alpha-1,2-mannosyltransferase*
241953	1.08	4.29E−03	GT15	Alpha-1,2-mannosyltransferase*
97566	1.90	2.59E−03	CE4	Chitin deacetylase**
180937	1.78	7.78E−06	GH18|CBM19	Chitinase**
221684	1.70	2.61E−05	GH16	None
209732	1.65	6.41E−06	GH20	Hexosaminidase**
241802	1.65	2.30E−06	GH81	Endo-1,3-beta-glucanase**
263075	1.60	1.27E−03	GH16	Xylanase/beta(1,4) glucanase**
204754	1.52	5.01E−04	GT2	Chitin synthase*
261112	1.57	1.31E−07	CE4	Chitin deacetylase**
11846	1.55	1.19E−04	GH18	Chitinase**
81356	1.54	7.78E−06	GH47	Alpha-1,2-mannosidase**
230502	1.53	8.72E−05	GH9	Endoglucanase**
222744	1.45	2.16E−04	GH72|CBM43	Glucanosyltransferase*
198772	1.44	2.42E−04	CE4	Chitin deacetylase**
219382	1.35	4.37E−03	GH3	Beta-glucosidase**
149043	1.33	4.99E−04	GH18	Chitinase**
181330	1.32	9.27E−04	CE4	Chitin deacetylase**
212115	0.83	8.21E−03	CE4	Chitin deacetylase**
258608	−0.97	7.17E−03	GH45|CBM1	Endoglucanase**
207865	−1.41	4.69E−03	GH45|CBM1	Endoglucanase**

a Log_2_ FC, log_2_ fold change. Positive log_2_ FC values denote upregulated genes, and negative log_2_ FC values denote downregulated genes.

b FDR, false discovery rate.

c Annotations obtained though CAZYmes Analysis Toolkit (109) are indicated with an asterisk. Annotations obtained through the *myco*CLAP database (110) are indicated by two asterisks. The remaining annotations are from JGI.

10.1128/mBio.02088-20.1 FIG S1 GO categories enriched in the upregulated fraction of the DE nonhost genes in response to physical interaction with *Mycetohabitans* sp. B13. Download FIG S1, PDF file, 0.03 MB. Copyright © 2020 Lastovetsky et al. 2020 Lastovetsky et al. This content is distributed under the terms of the Creative Commons Attribution 4.0 International license.

Much like during the antagonistic interaction before contact (Table 1), we detected the overexpression of genes encoding septins and mannosyltransferases in the nonhost during physical contact with endobacteria (Table 3). Septins are guanosine-5-triphosphate-binding proteins functioning in cell wall stress, cytoskeleton organization, and control of hyphal growth and morphology (51). In mammalian cells, septins are capable of forming septin cages around intracellular bacterial pathogens (52). However, as *Mycetohabitans* does not enter the nonhost hyphae, it is difficult to speculate about the role of septins in the *Rm-Mycetohabitans* antagonism.

**(iv) Genes involved in ROS metabolism.** In the nonhost, we detected differential expression of genes capable of generating ROS and causing oxidative damage through prooxidant activity (Table 4). Two of the upregulated genes were homologs to yeast *OYE2/3*. In yeast, the OYE2-OYE3 complex is involved in sensitizing cells to oxidative damage and promotes ROS-mediated programmed cell death (53). Two laccase genes and a copper amine oxidase gene were also overexpressed, both involved in production of hydrogen peroxide (54, 55). Conversely, only a single GST gene, which could be involved in ROS detoxification, was upregulated. In contrast, in the host, we saw upregulation of genes involved in antioxidant defense, encoding catalases, GST, and a serine/threonine kinase (Table 4); the latter is a homolog to Saccharomyces cerevisiae *YAK1* (234989) overexpressed in yeast during oxidative stress (56). Additionally, a gene encoding the reactive nitrogen species (RNS) producing nitric oxide synthase was downregulated.

**TABLE 4:** ROS response genes DE in the host (ATCC 52813) and the nonhost (ATCC 11559) during physical interaction with *Mycetohabitans* sp. B13

Protein ID	Log_2_ FC^*a*^	FDR^*b*^	IPR ID	Annotation^*c*^
Host				
292375	3.99	4.43E−04	IPR002226	Catalase
237694	2.03	4.77E−13	IPR004045	Glutathione *S*-transferase
238090	1.49	1.00E−13	IPR002226	Catalase
287063	0.88	3.09E−03	IPR002016	Heme peroxidase, plant/fungal/bacterial
287063	0.88	3.09E−03	IPR002207	Plant ascorbate peroxidase
2966	−0.63	3.40E−03	IPR008254	Flavodoxin/nitric oxide synthase
237561	−0.78	3.30E−06	IPR002007	Heme peroxidase, animal
Nonhost				
193785	4.04	5.96E−05	IPR001155	NADH:flavin oxidoreductase/NADH oxidase, yeast OYE2/3 homolog
216648	2.27	7.56E−16	IPR004046	Glutathione *S*-transferase, C-terminal
223952	2.00	4.44E−05	IPR001117	Multicopper oxidase, type 1
224787	1.55	2.23E−04	IPR001155	NADH:flavin oxidoreductase/NADH oxidase, yeast OYE2/3 homolog
294344	1.24	2.69E−05	IPR015798	Copper amine oxidase
15570	1.20	8.81E−03	IPR001117	Multicopper oxidase, type 1
29967	0.89	1.25E−03	IPR002007	Heme peroxidase, animal

a Log_2_ FC, log_2_ fold change. Positive log_2_ FC values denote upregulated genes, and negative log_2_ FC values denote downregulated genes.

b FDR, false discovery rate.

c Annotations given were generated by JGI. Homology to yeast OYE2/3 genes was determined by OrthoMCL (111).

In plants and animals, ROS and RNS are well-known for their role in defense against microbial pathogens (57, 58). They also have a part in symbiosis establishment (59). During plant-microbe interactions, production of ROS, known as the oxidative burst, exhibits a biphasic pattern in response to pathogens or incompatible symbionts. This biphasic response involves a low-amplitude first phase, followed by a higher ROS accumulation in the second phase (58, 59). The second phase does not occur in response to compatible symbionts (59). In contrast to animals and plants, little is known about fungal defense strategies involving ROS. Specifically, induction of antioxidant-encoding genes in fungi challenged by antagonistic bacteria (60) and by isolated MAMPs (18) was interpreted as a mechanism for removal of ROS generated in response to bacterial antagonists (18). On the basis of observations from other study systems and our data from the *Rm-Mycetohabitans* interactions, we hypothesized that in response to antagonistic endobacteria, the nonhost mounted a potent oxidative burst, whereas in the host, interaction with mutualistic bacteria induced a transient oxidative burst, which was subsequently quenched by antioxidant defense genes.

### ROS production in fungi interacting with bacteria.

**FIG 2:**
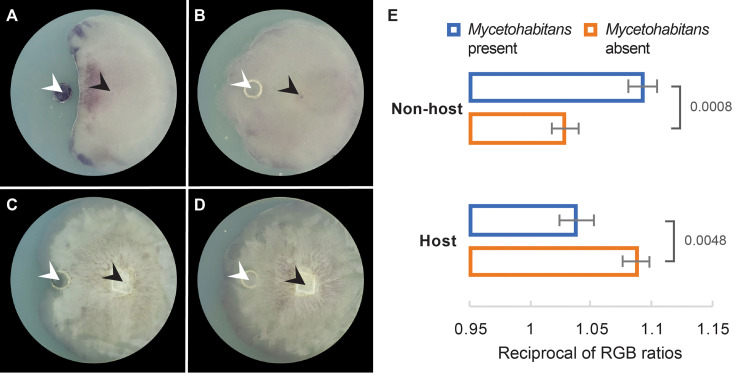
ROS output quantification in the nonhost *Rm* ATCC 11559 and the previously cured host *Rm* ATCC 52813 cocultivated with and without *Mycetohabitans* sp. B13 endobacteria isolated from the host ATCC 52813. (A) The nonhost interacting with *Mycetohabitans*. (B) The nonhost grown without *Mycetohabitans*. (C) The host interacting with *Mycetohabitans*. (D) The host grown without *Mycetohabitans*. In panels A to D, culture plates were stained with yellow, water-soluble NBT, which is reduced by superoxide radicals to blue, water-insoluble formazan, indicating ROS accumulation; white arrowheads show sites of *Mycetohabitans* or mock inoculation; black arrowheads point to sites of *Rm* inoculation. (E) Mean RGB color intensity ratios between portions of fungal colonies proximal and distal to the site of *Mycetohabitans* or mock inoculation. Means are the values from 9 or 10 replicate culture plates. Error bars indicate 1 standard error of the mean. *P* values from Student's *t* test are shown next to comparisons.

To test the hypothesis that ROS are accumulated by the nonhost and quenched by the host in response to *Mycetohabitans*, we quantified ROS produced by fungal colonies in close contact with bacteria (Fig. 2). Mycelia were stained with yellow, water-soluble nitroblue tetrazolium (NBT), which is reduced by superoxide radicals to blue, water-insoluble formazan (61). The resulting color distribution was analyzed by imaging software to determine changes in ROS accumulation. To standardize our measurements and minimize the impact of natural variation in ROS accumulation between replicate colonies, we measured the ratio of color intensity between the half of the fungal colony proximal to bacteria and the half that was distal. We found that ROS accumulation in the nonhost interacting with bacteria was significantly higher than in the nonhost growing alone (*P* < 0.001) (Fig. 2). In contrast, ROS accumulation in the host during physical contact with bacteria was significantly lower than in the host growing alone (*P* < 0.01). Together with gene expression analysis, these phenotypic results confirm the involvement of ROS in both the antagonistic and mutualistic bacterial-fungal interactions. During an antagonism, fungi are able sense bacterial presence and mount a defense response in the form of an oxidative burst. During a mutualistic interaction, the fungi actively quench ROS, allowing bacterial entry and symbiosis establishment.

### Precontact transcriptional responses in bacteria.

*Mycetohabitans* sp. B13 differentially expressed 219 genes in response to the nonhost (173 up- and 46 downregulated) and 123 genes in response to the host (107 up- and 16 downregulated), suggesting that bacteria were able to sense fungal presence and activated transcription in response to both fungi. Forty-seven genes were commonly upregulated in response to the nonhost and host.

**(i) Genes encoding secretion systems and their effectors.** Many of the genes commonly upregulated in response to both nonhost and host fungi encoded the type III secretion system (T3SS) machinery, candidate T3SS effector proteins, and a bacterial chitinase. T3SS is used by plant and animal pathogens to deliver protein effectors directly into the host cytoplasm for host manipulation (62). In the symbiosis between *Rm* and *M. rhizoxinica*, T3SS is crucial for the formation of the stable symbiotic association (46). In addition to components of the T3SS machinery itself, we identified 15 candidate effectors with a T3SS signal; over half of these are proteins of unknown function (Table S4). Remarkably, expression of these genes occurred before physical contact with the fungi, which is needed for translocation of the effectors through the T3SS. Another set of *M. rhizoxinica* factors known to enable symbiotic colonization of the *Rm* host comprises cell wall-degrading enzymes secreted through the type II secretion system, T2SS (23). T2SS translocates various proteins across the outer membrane of Gram-negative bacteria into the extracellular environment (63). The genomes of *M. rhizoxinica* (46) and *Mycetohabitans* sp. B13 encode a number of chitin-interacting proteins, including a chitinase, chitosinase, and a chitin-binding protein. Precontact, bacteria upregulated in response to both nonhost and host fungi the gene encoding chitinase, but not the other chitin-interacting genes or components of the T2SS machinery.

10.1128/mBio.02088-20.5 TABLE S4 Candidate T3SS effectors upregulated in *Mycetohabitans* sp. B13 during precontact and physical contact with the *Rm* host (ATCC 52813) and the *Rm* nonhost (ATCC 11559). Download Table S4, PDF file, 0.1 MB. Copyright © 2020 Lastovetsky et al. 2020 Lastovetsky et al. This content is distributed under the terms of the Creative Commons Attribution 4.0 International license.

Taken together, precontact bacterial responses to fungi involved upregulating expression of genes with known function in symbiosis establishment, indicating that symbionts were already primed for infection. Remarkably, bacteria upregulated the same symbiosis genes in response to nonhost and host, suggesting that they are equally equipped to infect both fungi.

### Physical contact transcriptional responses in bacteria.

During physical interaction with host and nonhost fungi, many more bacterial genes were DE compared to the precontact interaction. A total of 1,119 genes were DE in response to the nonhost and 453 genes in response to the host. There was an overlap of 253 genes that were commonly upregulated in response to both fungi and 92 were commonly downregulated. Since some of the DE bacterial genes, such as those encoding the T3SS cluster, components of the T2SS, and known T2SS effectors chitinase and chitosinase, have been experimentally validated as symbiosis factors in the *Rm-Mycetohabitans* mutualism (23, 26, 27), we hypothesize that other bacterial genes DE at this stage play a similar role. They include candidate T3SS effector genes, exopolysaccharide biosynthesis genes, genes encoding the Tol-Pal system, and response to oxidative stress genes.

**(i) Genes encoding secretion systems and their effectors.** Fifty-nine candidate T3SS effectors were overexpressed during physical interaction with both fungi (Table S4). Over a third of these encoded proteins of unknown function. Two candidate effectors had DNA-binding domains with homology to transcription factors, whereas three were predicted transporters. Two candidate effectors contained protein-interacting domains, leucine rich repeat (LRR) and F-box-like, and showed no homology to any bacterial proteins based on BLAST (64) searches of the NCBI databases, but were instead closer to eukaryotic proteins. LRR and F-box proteins are known virulence effectors in pathogenic bacteria (65, 66). LRR effectors block MAPK signaling through mimicking of eukaryotic E3 ubiquitin ligases (65), whereas F-box effectors mimic components of the eukaryotic protein degradation and regulation machinery (66). Another notable T3SS effector (*btl*13-19, gene ID 2599765431) was the *Mycetohabitans* transcription-activator-like (TAL)-like effector, Btl, that was overexpressed during interaction with both fungi. TAL effectors are found in plant-pathogenic *Xanthomonas* and *Ralstonia*, with homologs encoded in *Mycetohabitans* genomes (67). TAL effectors localize to plant host nuclei, where they bind specific DNA sequences through their central repeat DNA-binding domain and activate transcription of plant genes that facilitate infection (68). The *Mycetohabitans* sp. B13 Btl effector was recently shown to be involved in symbiosis by increasing fungal tolerance to cell membrane stress (69). Btl is expressed endohyphally, becomes translocated through the T3SS, and localizes to fungal nuclei (69), where it is capable of binding DNA with the same code as *Xanthomonas* TAL effectors (67).

Lastly, as expected, we detected the upregulation of genes encoding components of the T2SS, which contributes to *M. rhizoxinica*’s ability to infect its host (23). We also observed differential expression of genes encoding its known effectors, chitinase and chitosinase, which were upregulated, and the chitin-binding protein, which was downregulated. Downregulation of the gene for chitin-binding protein was unexpected, as this protein was previously shown to be upregulated during host-bacterium incubation in liquid (23), but this might be explained by the fact that during interaction on solid media, bacteria may not require the chitin-binding protein for attachment to fungal hyphae.

**(ii) Exopolysaccharide genes.** The upregulation of genes involved in the biosynthesis of exopolysaccharides (EPSs) during physical contact also suggested their involvement in the interaction with fungi. EPSs create a protective barrier around bacterial cells and play key roles in both pathogenesis (70) and symbiosis (71). For example, EPS mutants of mutualistic Sinorhizobium meliloti and Rhizobium leguminosarum are impaired in their ability to initiate symbiosis with the legume host (72, 73). In contrast, EPS mutants of *M. rhizoxinica* appear to be able to establish symbiosis with the *Rm* host in liquid culture (74). However, in our data set, genes encoding all components of the EPS biosynthesis cluster were extremely upregulated (>30-fold) due to physical contact with both nonhost and host, making it difficult to dismiss their role in interaction with fungi. It is thus possible that EPS is more important for interaction with fungi on solid media compared to liquid culture.

**(iii) Outer membrane stability and lipopolysaccharide genes.** The bacterial Tol-Pal system forms an inner-outer membrane-spanning complex that is well conserved in Gram-negative bacteria (75). It functions in maintaining outer membrane stability (76), cell division (77), resistance to detergents and bile salts (78), surface expression of the lipopolysaccharide (LPS) O antigen (79) and virulence (78). *Mycetohabitans* endobacteria encode the complete Tol-Pal system, with genes (2599765595 to 2599765598 and 2599765600) upregulated during physical interaction with nonhost and host fungi. Because this system is important in maintaining the stability of the bacterial cell envelope, it could contribute to survival in the intrahost environment, where changes in osmolarity and oxidative stress are likely encountered. In addition, we saw the upregulation of a specific sigma factor gene, *rpoE* (2599763969), along with a gene encoding its regulator, anti-sigma E protein RseA (2599763968), which in other bacteria regulate the envelope stress response (80). RpoE is required for intrahost survival of Salmonella enterica serotype Typhimurium (80), where it controls transcription of genes of the Tol-Pal system as well as many others involved in outer membrane biogenesis (81). Taken together, we hypothesize that during contact with fungi, endobacteria are faced with cell envelope stress and activate the envelope stress response, mediated by the specific sigma factor RpoE.

The outer membrane of Gram-negative bacteria is decorated with an LPS layer, composed of lipid A, core oligosaccharide, and O antigen. Eukaryotic hosts are able to recognize components of bacterial LPS, which plays important roles in pathogenic and symbiotic interactions (82). The *M. rhizoxinica* O antigen is involved in symbiosis establishment (26). Specifically, deletion of the O-antigen ligase gene, *waaL*, reduces the infection success rate and compromises intrahost proliferation of endosymbionts (26). The O antigen of *M. rhizoxinica* is a homopolymer of d-galactofuranose, which is also a common component of some filamentous fungal cell walls, leading to speculations that the O antigen could either act as a symbiosis factor recognized by the host to facilitate symbiosis establishment or, alternatively, as a cloaking device that shields the endobacteria by mimicking fungal cell components (26). Contrary to our expectations, we did not detect any differential expression of O antigen or LPS biosynthetic genes during physical interaction with host fungi, indicating that the O antigen is not synthesized specifically in response to the host but rather constitutively expressed. On the other hand, we detected significant downregulation of *waaL* (2599765323) during bacterial interaction with the nonhost. This pattern suggests that the O antigen is recognized by the fungi and either acts as a symbiosis signal during the mutualistic interaction with the host or activates fungal defenses during the antagonistic interaction with the nonhost.

**(iv) Genes involved in response to oxidative stress.** Fungal responses to endobacteria during physical contact revealed the involvement of ROS, with ROS amplification in the nonhost and quenching in the host (Table 4 and Fig. 2). In turn, bacterial gene expression reflected a response to ROS during physical interaction with both fungi (Table S5). Importantly, as bacteria are known to react to oxidative stress at the transcriptional level (57), this response appeared stronger during interaction with the nonhost. Only a single gene encoding GST (2599764292) was upregulated in response to both fungi. Bacterial GSTs have a role in detoxification of xenobiotics and in protection from oxidative stress through their ability to conjugate a large number of substrates to glutathione (83). In response to the nonhost only, we observed the upregulation of another GST gene (2599764292), as well as a gene encoding a glutathione synthase (2599765053), an enzyme responsible for the production of glutathione. Additionally, we saw the upregulation of another bacterial gene encoding antioxidant thioredoxin (2599763895) and a gene encoding coenzyme A pyrophosphatase (2599764015), which is known to catalyze the elimination of oxidized inactive CoA, and thus prevent the inhibition of CoA-utilizing enzymes as a result of exposure to ROS (84). Lastly, we saw overexpression of a gene encoding endonuclease III (2599765412) in response to nonhost only. Endonuclease III is part of the base excision repair mechanism that removes damaged pyrimidines (85). Base excision repair becomes activated in response to damage caused by oxygen radicals and constitutes an important mechanism of oxidative stress tolerance. A number of ROS-related genes were also downregulated in response to nonhost, encoding a superoxide dismutase (2599764121), which produces H_2_O_2_ from superoxide radicals, a peroxidase (2599762957), which converts H_2_O_2_ to H_2_O, and another GST (2599765091). Both up- and downregulation of ROS-related genes reflects a dynamic response to oxidative stress that was likely encountered by bacteria interacting with the nonhost fungi. Overall, consistent with our observation that bacteria encounter a ROS challenge during interaction with nonhost fungi, we identified a larger number of ROS-related genes DE by bacteria in the antagonistic interaction.

10.1128/mBio.02088-20.6 TABLE S5 DE *Mycetohabitans* sp. B13 genes involved in ROS detoxification during physical interaction with host *Rm* ATCC 52813 and nonhost *Rm* ATCC 11559 fungi. Download Table S5, PDF file, 0.03 MB. Copyright © 2020 Lastovetsky et al. 2020 Lastovetsky et al. This content is distributed under the terms of the Creative Commons Attribution 4.0 International license.

### Conservation of bacterial genes involved in interaction with fungi.

Little is known about the molecular underpinnings governing bacterial interaction with fungi beyond the characterized symbiosis factors of *M. rhizoxinica* (23, 26, 27). To learn about the conservation of genes involved in interaction with and response to fungi in *Mycetohabitans* endobacteria, we performed orthologous clustering of protein sequences across 20 *Burkholderia* species (closest free-living relatives of *Mycetohabitans*) with different lifestyles that ranged from soil inhabitants to human pathogens (Table S6). We included three genomes of *Mycetohabitans* endosymbionts of *Rm*, *M. rhizoxinica* HKI 454 (29), *Mycetohabitans* sp. B13, and *Mycetohabitans* sp. B14 (NCBI accession number PRJNA303197). None of the genes encoding known endobacteria symbiosis factors (T3SS, T2SS, chitinase, chitosinase, and LPS) were found exclusively in the genomes of endofungal bacteria. Moreover, the majority of fungus-interacting genes identified from our transcriptional profiling (Tol-Pal, EPS, ROS response) were also not exclusive to *Mycetohabitans*. This observation suggests that the ability to engage in symbioses with fungi is largely facilitated by a preexisting gene repertoire found in *Burkholderia* of diverse lifestyles. For example, a homolog of the T2SS effector chitinase, which facilitates endobacterial entry into host hyphae (23), is also encoded in the genomes of human-pathogenic Burkholderia cenocepacia J2315 and AU105, B. mallei ATCC 23344, B. pseudomallei BPC006 as well as a soil saprotroph B. thailandensis E264. Analogously, homologs of genes in the *M. rhizoxinica* T3SS cluster, which is required for successful symbiosis establishment (27), are encoded in over half of the examined *Burkholderia* genomes. Conversely, 35 of the genes commonly upregulated during physical interaction with host and nonhost fungi were exclusively encoded in genomes of *Mycetohabitans* (Table S7). Almost half of these genes encoded candidate T3SS effectors and included *btl19-13* (86), as well as the candidate LRR- and F-box-domain effectors identified in this study. T3SS effector repertoire of pathogenic bacteria is known to shape their host range and can be extremely variable even among closely related strains (87). It is thus possible that the T3SS effector repertoire of *Mycetohabitans* evolved for exclusive interaction with fungi.

10.1128/mBio.02088-20.7 TABLE S6 *Mycetohabitans* and *Burkholderia* species and strains used in the OrthoMCL clustering analysis. Download Table S6, PDF file, 0.05 MB. Copyright © 2020 Lastovetsky et al. 2020 Lastovetsky et al. This content is distributed under the terms of the Creative Commons Attribution 4.0 International license.

10.1128/mBio.02088-20.8 TABLE S7 *Mycetohabitans* sp. B13 genes carried exclusively in the genomes of endofungal bacteria and upregulated during physical contact with host and nonhost fungi. Download Table S7, PDF file, 0.04 MB. Copyright © 2020 Lastovetsky et al. 2020 Lastovetsky et al. This content is distributed under the terms of the Creative Commons Attribution 4.0 International license.

### Ecology of Mucoromycotina interactions with endobacteria.

The antagonistic response of nonhost *Rm* to *Mycetohabitans* isolated from the host *Rm* is shared by other Mucoromycotina, such as Rhizopus oryzae and Mucor circinelloides, which do not become colonized by these endobacteria (24). To assess the incidence of *Mycetohabitans* endobacteria across Mucoromycotina, we screened a collection of over 60 representatives of the Mucoromycotina genera, such as *Absidia*, *Cokeromyces*, *Cunninghamella*, *Gongronella*, *Mucor*, *Mycotypha*, *Phycomyces*, *Radiomyces*, *Rhizopus*, *Syncephalastrum*, *Thamnidium*, *Umbelopsis*, and *Zygorhynchus*, with a particular focus on *Rhizopus*, represented by over 40 isolates (Table S8). With the exception of several isolates of *Rm*, *Mycetohabitans* endobacteria were not detected in any of the isolates examined. This observation is somewhat surprising, as Mucoromycotina are aggressively growing saprotrophs that likely interact with diverse bacteria. Moreover, unlike ascomycetes, such as *Aspergillus* and *Penicillium*, which share a similar lifestyle and produce a wide range of antibacterial secondary metabolites in response to bacteria (88, 89), Mucoromycotina possess only a limited repertoire of secondary metabolite gene clusters (Table S9). In fact, in *Rm* only a single candidate secondary metabolism gene (nonribosomal peptide synthase-like; nonhost protein ID 294600, host protein ID 212705) was upregulated in response to physical interaction with bacteria in both nonhost and host fungi, whereas a nonribosomal peptide synthase gene (290813) was downregulated in the host. On the basis of these patterns, we speculate that the incidence of endobacteria across Mucoromycotina is controlled by the nonhost ability to detoxify antifungal metabolites, remodel the cell wall structure, and mount a potent ROS burst to prevent chitinase-mediated bacterial entry rather than by profuse biosynthesis of antibacterial secondary metabolites.

10.1128/mBio.02088-20.9 TABLE S8 Mucoromycotina isolates screened for the presence of endobacteria. Download Table S8, PDF file, 0.04 MB. Copyright © 2020 Lastovetsky et al. 2020 Lastovetsky et al. This content is distributed under the terms of the Creative Commons Attribution 4.0 International license.

10.1128/mBio.02088-20.10 TABLE S9 Secondary metabolite gene (SMG) clusters in genomes of Eurotiomycetes (Ascomycota) and Mucoromycotina. Download Table S9, PDF file, 0.3 MB. Copyright © 2020 Lastovetsky et al. 2020 Lastovetsky et al. This content is distributed under the terms of the Creative Commons Attribution 4.0 International license.

### Conclusion.

**FIG 3:**
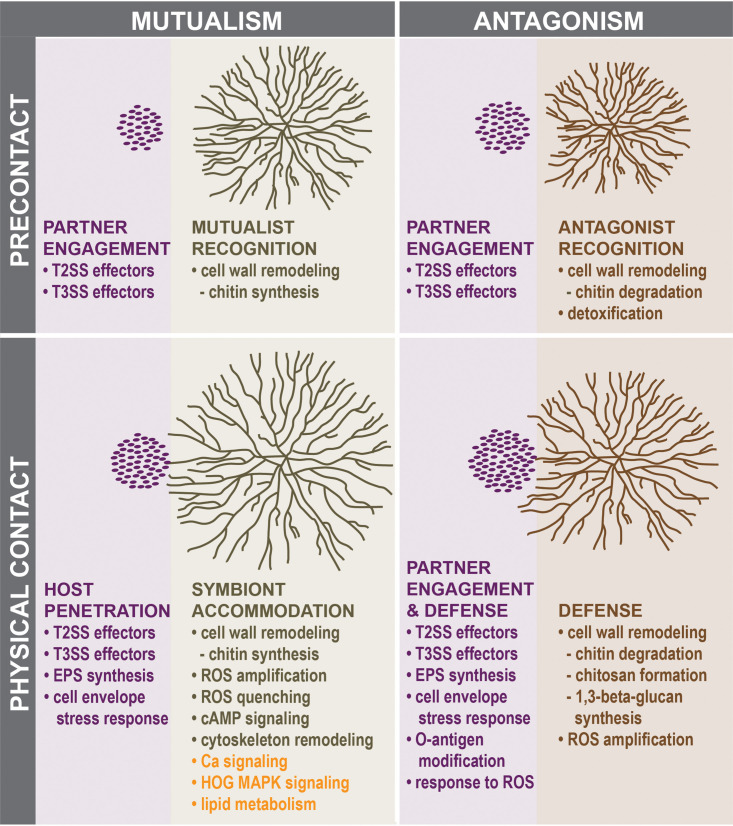
Hypotheses describing molecular dialogues between early divergent fungi and bacteria in a mutualism versus an antagonism before and after physical contact. The presented hypotheses were formulated on the basis of changes in gene expression patterns described in this study and by Lastovetsky et al. (24), shown in orange. Bacterial cells are represented as purple ovals, and fungal mycelia are depicted in green (host) and brown (nonhost).

Using transcriptional profiling, we deciphered molecular dialogues between early divergent Mucoromycotina fungi and *Mycetohabitans* bacteria in an antagonism and mutualism at two time points, precontact and contact (Fig. 3). Precontact, the antagonistic interaction involved the apparent detoxification of an unknown bacterial compound by the nonhost, whereas the mutualistic interaction elicited a weak response from the host. Commonalities in the fungal responses during contact centered around expression of genes involved in cell wall modification and ROS metabolism. These patterns pointed to specific alterations to the fungal cell wall and to an increase in ROS production during the antagonistic interaction, which we confirmed with ROS measurements. In addition to confirming differential ROS responses in the establishment of fungal-bacterial mutualisms and antagonisms, these phenotypic observations demonstrated the predictive power of the framework for interactions between early divergent Mucoromycotina fungi and bacteria proposed here. We hypothesize that contact responses mounted by the nonhost contributed to its ability to resist bacterial invasion, a behavior shared by other Mucoromycotina (24). In contrast, the mutualistic interaction, in addition to the involvement of fungal genes encoding the HOG MAPK signaling pathway and lipid metabolism described previously (24), was marked by transcriptional upregulation of genes in the cAMP signaling pathway and cytoskeletal rearrangements, thus revealing a set of fungal genes formerly unknown for their role in symbiosis establishment. Remarkably, precontact transcriptomic changes indicated that bacteria did not discriminate between host and nonhost fungi and that they appeared to be equally equipped to infect both. During physical contact with fungi, in addition to factors specific to the *Rm-Mycetohabitans* symbiosis, endobacteria engaged a common set of factors used to interact with other eukaryotic hosts. Overall, our findings contribute to building a conceptual framework for understanding the molecular factors mediating fungal-bacterial interactions.

## MATERIALS AND METHODS

### Strains, culture conditions, removal and extraction of *Mycetohabitans* endobacteria.

Rhizopus microsporus (*Rm*) strains ATCC 52813 and ATCC 11559 were cultivated on potato dextrose agar (PDA) at 30°C. *Rm* ATCC 52813 was cured of its endobacteria as previously described (21). Endobacteria were extracted from 3-day-old host mycelium ground in 800 μl of Luria-Bertani (LB) broth using a plastic mortar and centrifuged at 4,000 × *g* for 2 min. The supernatant was passed twice through a 2-μm Whatman filter, plated onto LB agar plates supplemented with 1% glycerol, and incubated at 30°C.

### *Mycetohabitans* sp. strain B13 genome sequencing, assembly, and annotation.

The draft genome of *Mycetohabitans* sp. B13 was generated at the U.S. Department of Energy Joint Genome Institute (DOE JGI) using the Pacific Biosciences (PacBio) sequencing technology (90). A PacBio SMRTbell library was constructed and sequenced on the PacBio RS platform, which generated 144,221 filtered subreads totaling 586.4 Mbp. All general aspects of library construction and sequencing performed at the JGI can be found at http://www.jgi.doe.gov. The raw reads were assembled using HGAP v. 2.2.0.p1 (91). The final draft assembly contained two contigs in two scaffolds, totaling 3.6 Mbp in size. The input read coverage was 98.4×. Genes were identified using Prodigal (92), followed by a round of manual curation using GenePRIMP (93) for finished genomes and draft genomes in fewer than 10 scaffolds. The predicted coding DNA sequences (CDSs) were translated and used to search the National Center for Biotechnology Information (NCBI) nonredundant database, UniProt, TIGRFam, Pfam, KEGG, COG, and InterPro databases. The tRNAScan-SE tool (94) was used to find tRNA genes, whereas rRNA genes were identified by searches against models of the rRNA genes built from SILVA (95). Additional gene prediction analysis and manual functional annotation was performed within the Integrated Microbial Genomes (IMG) platform (96). The whole-genome average nucleotide identity (ANI) between the genomes of endofungal *Mycetohabitans* was computed using the ANI calculator available at http://enve-omics.ce.gatech.edu/ani/index.

### RNA-seq experiment and data analysis.

We examined five different conditions at two different time points: (i) cured *Rm* host (ATCC 52813) cultured alone, (ii) cured ATCC 52813 cultured with *Mycetohabitans* sp. B13, (iii) nonhost *Rm* (ATCC 11559) cultured alone, (iv) ATCC 11559 cultured with *Mycetohabitans* sp. B13, and (v) *Mycetohabitans* sp. B13 cultured alone. The two time points corresponded to interaction at a distance (precontact) and interaction during physical contact. During precontact interaction, which occurred 50 h postinoculation of ATCC 52813 and 56 h postinoculation of ATCC 11559 due to naturally different growth rates, fungi have not yet physically contacted the site of bacterial inoculation. During physical interaction, at 67 h postinoculation of ATCC 52813 and 93 h postinoculation of ATCC 11559, fungi have just come into contact with the site of bacterial inoculation. For each condition, bacteria were inoculated on an LB plus 1% glycerol (LB + 1% glycerol) agar plug on one side of a half-strength PDA plate and an ∼0.5-cm^2^ mat of fungal mycelium placed ∼2 cm away. Plates were incubated at 30°C. Fungal mycelium was harvested from the interaction zone, and bacterial cells were scraped off the agar. Each condition had three biological replicates, each consisting of three culture plates pooled prior to RNA extraction. Total RNA was extracted with the Ambion ToTALLY Total RNA isolation kit (Life Technologies), and rRNA was removed with RiboZero Magnetic Gold kit (Epicentre). RNA sequencing libraries were prepared using the NEBNext mRNA Library Prep Reagent Set for Illumina and sequenced at the Cornell University Biotechnology Resource Center using the Illumina Hi-Seq 100-bp paired-end platform. Illumina data were quality controlled using the FASTX-Toolkit (97), and the reads were mapped onto either ATCC 52813 or ATCC 11559 genomes using TopHat (98). Transcript abundances were quantified with CuffDiff (98), and differential gene expression analysis was performed with EdgeR (99). The false discovery rate (FDR) value of 0.01 was used as a cutoff for the identification of differentially expressed genes.

### GO category functional enrichment analysis.

GO annotation for all the genes from the ATCC 11559 genome were obtained from JGI Mycocosm (100) and imported into Blast2GO (101). Functional enrichment analysis was performed using a Fisher’s exact test with a *P* value cutoff of 0.01 on all the upregulated genes in response to interaction with bacteria.

### Manual annotation of cytoskeleton-related genes.

The differentially expressed (DE) cytoskeleton-related genes were manually annotated based on KOG (eukaryotic orthologous groups) annotations in the “cytoskeleton” class as well as based on involvement in cytoskeleton-related function. For example, Rho GTPase-activating proteins (IPR000198) are known to control actin cytoskeletal formation. This list of genes was further validated and annotated with PSI-BLAST (102) searches of Swiss-Prot/UniProt databases according to homology to known cytoskeleton-related genes.

### ROS visualization.

Host *Rm* ATCC 52813 and nonhost *Rm* ATCC 11559 were cultivated with and without *Mycetohabitans* sp. B13 on LB plates supplemented with 1% glycerol. LB + 1% glycerol medium was used instead of PDA to limit sporulation in *Rm* ATCC 11559, as dark-colored sporangiospores interfere with ROS visualization; the cured host *Rm* ATCC 52813 does not produce spores. One side of the plate was inoculated with 2 μl of 5 × 10^5^ ml^−1^ suspension of nonhost *Rm* ATCC 11559 spores or a 0.81-cm^2^ square of cured host *Rm* ATCC 52813 mycelium. To prepare bacteria for inoculation, multiple colonies were transferred to 0.8 ml of LB + 1% glycerol broth and vortexed to form a dense suspension. Ten microliters of bacterial suspension was placed 2 cm away from the site of fungal inoculation onto an outlined area made by puncturing the agar with the back of a sterile 1,000-μl pipette tip. For no-bacterium controls (mock inoculations), plugs were formed but not inoculated. Plates were left uncovered for 10 min to allow any liquid solutions to dry and incubated at 29°C. Due to differing growth rates between the nonhost and host fungi, staining was performed at a time point when fungi were just coming into contact with bacteria to mimic the physical contact time point of RNA-seq analysis, 52 h postinoculation in the nonhost and 67 h postinoculation in the host fungi.

For visualization of ROS, the NBT stain was prepared from 2.5 mM NBT (AstaTech, Inc., lot P102-15519) and 5 mM (*N*-morpholino) propane sulfonate-NaOH buffer (pH 7.6) (103). The solution was shielded from light to prevent photo-oxidation. Plates were flooded with 2 ml of NBT stain that was applied directly onto the mycelium and immediately spread with a glass spreader to ensure penetration through the hydrophobic exterior. Plates were imaged 10 min later in a lightbox with a Samsung Galaxy S9 camera calibrated to a white balancer reference card. Images were analyzed in ImageJ using the Color Histogram plugin (104). The entire colony on each plate was selected. Areas proximal and distal to bacterial inoculation site were designated using the midpoint of the fungal inoculant as a dividing line between the sides. Area and mean red, green, and blue pixel values were recorded for each selected side using the Color Histogram tool. To account for ROS produced by *Mycetohabitans*, we included the entirety of the bacterial plug area to proximal mycelium selections that reached the plug and removed the average area-weighted contribution of bacterial plug red, green, and blue intensity (RGB) values from the RGB values of these selections. Finally, in order to reduce the impact of natural variation in ROS production between plates, the ratio of proximal to distal side was calculated for each replicate. Relationships between these ratios were evaluated using the Student’s *t* test.

### T3SS effector identification.

Effective T3 v. 2.0.1 (105), through https://effectors.csb.univie.ac.at, accessed on 26 May 2020 was used to scan the amino acid sequences of all differentially expressed genes in *Mycetohabitans* endobacteria during interaction with host and nonhost fungi. A cutoff score of 0.9999 was used to classify “secreted” protein. EffectiveELD with a minimal z-score of 4 was used for prediction of eukaryotic-like domains.

### Identification of orthologs in the genomes of *Mycetohabitans* and *Burkholderia*.

We collected amino acid sequences for all protein-coding genes from *Mycetohabitans* and *Burkholderia* genomes listed in Table S5 in the supplemental material and conducted an all-versus-all BLASTp (64) search with parameters: E-value cutoff = 1 × 10^−3^ and maximum matches = 500. OrthoMCL (106) was used to identify orthologs with parameters: mode = 3, pi_cutoff = 0, pv_cutoff = 1 × 10^−3^, and inflation = 0.

### Detection of endobacteria in Mucoromycotina.

Fungal isolates were obtained from the American Research Service Culture Collection (NRRL), American Type Culture Collection (ATCC), Centraalbureau voor Schimmelcultures (CBS), French National Reference Center for Invasive Mycoses and Antifungals (CNRMA), Cornell University Plant Pathology & Plant-Microbe Biology Fungal Culture Collection (CU), Duke University (DUKE), and Jena Microbial Resource Collection (FSU). They were sampled for DNA by scraping a small amount of frozen glycerol stock and placing it in 9 μl Sample Buffer of the illustra GenomiPhi V2 whole-genome amplification kit (GE LifeSciences). Sample DNA was then globally amplified in accordance with the manufacturer’s protocol. All GenomiPhi products were diluted 1:20 in molecular biology grade water (HyClone) in preparation for PCR. PCR was conducted to confirm fungal identity with LR1 and NDL22 primers (107) and to detect the presence of *Burkholderia* endobacteria with GlomGiGf and LSU483r primers (108). Purified PCR products were sequenced using BigDye Terminator v3.1 chemistry (Applied Biosystems). Each reaction mixture contained 3.5 μl molecular biology grade water (HyClone), 2.5 μl 5BigDye X Sequencing Buffer, 1 μl forward or reverse primer, 0.5 μl 5 M betaine, and 0.05 μl BigDye Premix. The cycling conditions were in accordance with those described in the BigDye v3.1 manual. DNA was filtered and extracted using 70 M Sephadex columns (GE Healthcare) and sequenced by the Cornell University Life Sciences Core Laboratory Center on the ABI 3730xl sequence analyzer.

### Accession number(s).

The whole-genome shotgun *Mycetohabitans* sp. B13 project is deposited at GenBank under accession numbers FTPM01000001 to FTPM01000002. The transcriptome data are available at the NCBI GEO database under the accession number GSE98095.
