# Supplementary material for: Molecular Dialogues between Early Divergent Fungi and Bacteria in an Antagonism versus a Mutualism
Source: mBio. 2020 Sep 8;11(5):e02088-20. doi: 10.1128/mBio.02088-20 (PMC7482071; doi:10.1128/mBio.02088-20)
Supplement: TABLE S1 [file mBio.02088-20-st001.pdf]

Table S1. Genome statistics for *Mycetohabitans* sp. B13 and *Mycetohabitans* sp. B14 as well as previously sequenced *Mycetohabitans* included for comparison.

	***Mycetohabitans* sp. B13**	***Mycetohabitans* sp. B14**	***M. rhizoxinica* HKI454**	***M. endofungorum* HKI 456**
**DNA, total number of bases**	3579262	3653645	3750138	328408
**DNA coding number of bases**	87.50%	87.08%	88.73%	85.75%
**DNA G+C number of bases**	60.59%	60.56%	60.71%	61.27%
**DNA scaffolds**	2	3	3	76
**Genes total number**	3292	3465	3938	3051
**Protein coding genes**	3219	3394	3870	2631
**Pseudo Genes**	404	417	NA	369
**RNA genes**	73	71	68	62
**rRNA genes**	9	9	9	5
**5S rRNA**	3	3	3	3
**16S rRNA**	3	3	3	1
**23S rRNA**	3	3	3	1
**tRNA genes**	47	47	59	43
**Other RNA genes**	17	15	NA	5
**Reference**	This study	This study	(1)	(2)
**GenBank accession number**	FTPM01000000	FTPJ01000000	FR687359<br>FR687360<br>FR687361	GCA_002927045
**Fungal host**	ATCC 52813	ATCC 52814	ATCC 62417	CBS 112285

1. Lackner G, Moebius N, Partida-Martinez L, Hertweck C. 2011. Complete genome sequence of *Burkholderia rhizoxinica*, an endosymbiont of *Rhizopus microsporus*. Journal of Bacteriology 193:783-784.
2. Estrada-de Los Santos P, Palmer M, Chavez-Ramirez B, Beukes C, Steenkamp ET, Briscoe L, Khan N, Maluk M, Lafos M, Humm E, Arrabit M, Crook M, Gross E, Simon MF, Dos Reis Junior FB, Whitman WB, Shapiro N, Poole PS, Hirsch AM, Venter SN, James EK. 2018. Whole genome analyses suggests that *Burkholderia* sensu lato contains two additional novel genera (*Mycetohabitans* gen. nov., and *Trinickia* gen. nov.): Implications for the evolution of diazotrophy and nodulation in the Burkholderiaceae. Genes 9:389.
