# Supplementary material for: Molecular Dialogues between Early Divergent Fungi and Bacteria in an Antagonism versus a Mutualism
Source: mBio. 2020 Sep 8;11(5):e02088-20. doi: 10.1128/mBio.02088-20 (PMC7482071; doi:10.1128/mBio.02088-20)
Supplement: TABLE S2 [file mBio.02088-20-st002.pdf]

Table S2. Signaling genes DE in the *Rm* host fungi (ATCC 52813) during physical interaction with endobacteria. Log_2FC, log_2 fold change; positive Log_2FC values denote upregulated genes, negative Log_2FC values denote downregulated genes; FDR, false discovery rate.

Protein ID	Log_2FC	FDR	IPR ID	IPR domain	KOG ID	KOG annotation	KOG class	GO ID	GO category
315343	3.78	2.47E-05	IPR001206	Diacylglycerol kinase	KOG1169	Diacylglycerol kinase	Lipid transport and metabolism	GO:0007205	protein kinase C activation
252161	2.65	1.07E-07	IPR000198	RhoGAP	KOG2710	Rho GTPase-activating protein	Signal transduction mechanisms	GO:0007165	signal transduction
60967	2.56	6.10E-04	IPR000219	Dbl homology (DH) domain	KOG4305	RhoGEF GTPase	Signal transduction mechanisms	GO:0035023	regulation of Rho protein signal transduction
249145	2.06	1.19E-09	IPR001206	Diacylglycerol kinase	KOG1169	Diacylglycerol kinase	Lipid transport and metabolism	GO:0007205	activation of protein kinase C activity
136105	2.00	2.07E-08	IPR001806	Ras GTPase	KOG0393	Ras-related small GTPase, Rho type	General function prediction only	GO:0007264	small GTPase mediated signal transduction
264384	1.97	2.88E-13	none	no domains	KOG2058	Ypt/Rab GTPase activating protein	Intracellular trafficking, secretion, and vesicular transport	GO:0004308	exo-alpha-sialidase activity
185441	1.81	1.24E-06	IPR017442	Serine/threonine protein kinase-related	KOG0612	Rho-associated, coiled-coil containing protein kinase	Signal transduction mechanisms	GO:0006468	protein amino acid phosphorylation
271786	1.72	2.78E-04	IPR000219	Dbl homology (DH) domain	KOG4424	Predicted Rho/Rac guanine nucleotide exchange factor/faciogenital dysplasia protein 3	Signal transduction mechanisms	GO:0035023	regulation of Rho protein signal transduction
201417	1.41	4.72E-13	IPR001789 IPR003661 IPR013655 IPR013655 IPR001610	Signal transduction response regulator, receiver region; Signal transduction histidine kinase, subgroup 1, dimerisation and phosphoacceptor region; PAS fold-3.ATP-binding region, ATPase-like, PAC motif	KOG0519	Sensory transduction histidine kinase	Signal transduction mechanisms	GO:0007165	signal transduction

Protein ID	Log_2FC	FDR	IPR ID	IPR domain	KOG ID	KOG annotation	KOG class	GO ID	GO category
286008	1.22	3.15E-04	IPR002373	cAMP/cGMP-dependent protein kinase	KOG1113	cAMP-dependent protein kinase types I and II, regulatory subunit	Signal transduction mechanisms	GO:0006468	protein amino acid phosphorylation
274088	1.21	3.24E-05	IPR000198	RhoGAP	KOG4270	GTPase-activator protein	Signal transduction mechanisms	GO:0007165	signal transduction
266182	1.19	5.69E-04	IPR000719 IPR003096 IPR001715	Protein kinase, core, SM22/calponin, Calponin-like actin-binding	KOG0198	MEKK and related serine/threonine protein kinases	Signal transduction mechanisms	GO:0006468	protein amino acid phosphorylation
234989	1.16	1.06E-04	IPR017442	Serine/threonine protein kinase-related	KOG0667	Dual-specificity tyrosine-phosphorylation regulated kinase	General function prediction only	GO:0006468	protein amino acid phosphorylation
226218	1.01	4.85E-03	IPR001060 IPR000198	Cdc15/Fes/CIP4, RhoGAP	KOG1450	Predicted Rho GTPase-activating protein	Signal transduction mechanisms	GO:0007165	signal transduction
300418	1.00	3.11E-05	IPR000719 IPR008352	Protein kinase, core, MAP kinase, p38	KOG0660	Mitogen-activated protein kinase	Signal transduction mechanisms	GO:0006468	protein amino acid phosphorylation
235547	1.00	4.26E-07	IPR002219 IPR001452 IPR001060	Protein kinase C, phorbol ester/diacylglycerol binding, Src homology-3, Cdc15/Fes/CIP4	KOG3565	Cdc42-interacting protein CIP4	Cytoskeleton	GO:0007242	intracellular signaling cascade
278678	0.90	3.20E-03	IPR017442	Serine/threonine protein kinase-related	KOG0616	cAMP-dependent protein kinase catalytic subunit (PKA)	Signal transduction mechanisms	GO:0006468	protein amino acid phosphorylation
233858	0.85	1.89E-03	IPR001164	Arf GTPase activating protein	KOG0702	Predicted GTPase-activating protein	Signal transduction mechanisms	GO:0008060	ARF GTPase activator activity
277000	0.84	5.10E-03	IPR001849 IPR001164	Pleckstrin-like, Arf GTPase activating protein	KOG0521	Putative GTPase activating proteins (GAPs)	Signal transduction mechanisms	GO:0008060	ARF GTPase activator activity
202982	0.82	6.04E-04	IPR002498	Phosphatidylinositol-4-phosphate 5-kinase, core	KOG0229	Phosphatidylinositol-4-phosphate 5-kinase	Signal transduction mechanisms	GO:0046488	phosphatidylinositol metabolic process

Protein ID	Log_2FC	FDR	IPR ID	IPR domain	KOG ID	KOG annotation	KOG class	GO ID	GO category
270618	0.74	1.99E-03	IPR013079 IPR003094 IPR013078	Bifunctional 6-phosphofructo-2-kinase/fructose-2, 6-bisphosphate 2-phosphatase, Fructose-2,6-bisphosphatase, Phosphoglycerate mutase	KOG0234	Fructose-6-phosphate 2-kinase/fructose-2,6-biphosphatase	Carbohydrate transport and metabolism	GO:0006003	fructose 2,6-bisphosphate metabolic process
286014	0.72	4.26E-03	IPR000719 IPR008352	Protein kinase, core, MAP kinase, p38	KOG0660	Mitogen-activated protein kinase	Signal transduction mechanisms	GO:0006468	protein amino acid phosphorylation
236201	0.70	1.57E-04	IPR001199	Cytochrome b5	KOG4035	Coeffector of mDia Rho GTPase, regulates actin polymerization and cell adhesion turnover	Cytoskeleton	GO:0046914	transition metal ion binding
237596	0.69	4.25E-04	IPR000198	RhoGAP	KOG4270	GTPase-activator protein	Signal transduction mechanisms	GO:0007165	signal transduction
234830	0.66	9.11E-03	IPR002073 IPR011006	3'5'-cyclic nucleotide phosphor-diesterase, CheY-like	KOG3689	Cyclic nucleotide phosphodiesterase	Signal transduction mechanisms	GO:0007165	signal transduction
268827	0.65	2.36E-03	IPR003109	GoLoco				GO:0007165	signal transduction
250648	0.63	9.71E-03	IPR001806 IPR017441	Ras GTPase, Protein kinase ATP binding, conserved site	KOG4423	GTP-binding protein-like, RAS superfamily	Signal transduction mechanisms	GO:0007264	small GTPase mediated signal transduction
244189	0.50	5.10E-03	IPR002014 IPR000306 IPR003903	VHS, Zinc finger, FYVE-type, Ubiquitin interacting motif	KOG1818	Membrane trafficking and cell signaling protein HRS, contains VHS and FYVE domains	Signal transduction mechanisms	GO:0006886	intracellular protein transport
197380	-0.55	4.85E-04	IPR005946	Phosphoribosyl pyro-phosphokinase					

Protein ID	Log_2FC	FDR	IPR ID	IPR domain	KOG ID	KOG annotation	KOG class	GO ID	GO category
233491	-0.57	4.69E-04	IPR001048 IPR000706 IPR000534	Aspartate/glutamate/uridylate kinase, N-acetyl-gamma-glutamyl-phosphate reductase, Semialdehyde dehydrogenase, NAD-binding	KOG2436	Acetylglutamate kinase/acetylglutamate synthase	Amino acid transport and metabolism	GO:0006520	amino acid metabolic process
284787	-0.61	1.06E-03	IPR002290	Serine/threonine protein kinase	KOG3741	Poly(A) ribonuclease subunit	RNA processing and modification	GO:0006468	protein amino acid phosphorylation
236630	-0.63	6.23E-03	IPR001849 IPR000219 IPR000270 IPR001331	Pleckstrin-like, Dbl homology (DH) domain, Octicosapeptide/Phox/Bem1p, Guanine-nucleotide dissociation stimulator, CDC24, conserved site	KOG3519	Invasion-inducing protein TIAM1/CDC24 and related RhoGEF GTPases	Signal transduction mechanisms	GO:0005089	Rho guanyl-nucleotide exchange factor activity
284927	-0.69	8.96E-04	IPR016034	Phosphatidylinositol-4-phosphate 5-kinase, core, subgroup	KOG0229	Phosphatidylinositol-4-phosphate 5-kinase	Signal transduction mechanisms	GO:0046488	phosphatidylinositol metabolic process
200320	-0.76	6.26E-03	IPR000195	RabGAP/TBC	KOG2058	Ypt/Rab GTPase activating protein	Intracellular trafficking, secretion, and vesicular transport	GO:0005097	Rab GTPase activator activity
235463	-0.76	4.66E-03	IPR001895 IPR000651	Guanine-nucleotide dissociation stimulator CDC25, Guanine nucleotide exchange factor for Ras-like GTPases, N-terminal	KOG3417	Ras1 guanine nucleotide exchange factor	Signal transduction mechanisms	GO:0005085	guanyl-nucleotide exchange factor activity
15091	-0.81	3.17E-03	IPR008936	Rho GTPase activation protein				GO:0007165	signal transduction

Protein ID	Log$_2$FC	FDR	IPR ID	IPR domain	KOG ID	KOG annotation	KOG class	GO ID	GO category
88449	-0.81	1.17E-04	IPR000719	Protein kinase, core				GO:0004672	protein kinase activity
199697	-0.83	1.64E-04	IPR001048	Aspartate/ glutamate/ uridylate kinase	KOG0456	Aspartate kinase	Amino acid transport and metabolism	GO:0008652	amino acid biosynthetic process
313597	-0.83	9.83E-06	IPR000403 IPR016024	Phosphatidylinositol 3- and 4-kinase, catalytic, Armadillo-type fold	KOG0903	Phosphatidylinositol 4-kinase, involved in intracellular trafficking and secretion	Signal transduction mechanisms	GO:0016773	phosphotransferase activity, alcohol group as acceptor
222087	-1.27	7.48E-19	IPR017442	Serine/threonine protein kinase-related	KOG0590	Checkpoint kinase and related serine/threonine protein kinases	Cell cycle control, cell division, chromosome partitioning	GO:0006468	protein amino acid phosphorylation
