# Supplementary material for: Molecular Dialogues between Early Divergent Fungi and Bacteria in an Antagonism versus a Mutualism
Source: mBio. 2020 Sep 8;11(5):e02088-20. doi: 10.1128/mBio.02088-20 (PMC7482071; doi:10.1128/mBio.02088-20)
Supplement: TABLE S3 [file mBio.02088-20-st003.pdf]

Table S3. Cell cycle related genes DE in the host (ATCC 52813) during physical interaction with *Mycetohabitans* sp. B13. Log_2FC, log_2 fold change; positive Log_2FC values denote upregulated genes, negative Log_2FC values denote downregulated genes; false discovery rate, FDR.

Protein ID	Log_2FC	FDR	Interpro ID	Domain	KOG ID	KOG definition	KOG Class
232829	1.69	1.08E-07	IPR001060 IPR001452	Cdc15/Fes/CIP4, Src homology-3	KOG2398	Predicted proline-serine-threonine phosphatase-interacting protein (PSTPIP)	Cell cycle control, cell division, chromosome partitioning
286595	1.16	1.59E-11	IPR007632	Protein of unknown function DUF590	KOG2513	Protein required for meiotic chromosome segregation	Cell cycle control, cell division, chromosome partitioning
312571	1.00	6.81E-03	IPR001452 IPR001060	Src homology-3, Cdc15/Fes/CIP4	KOG2398	Predicted proline-serine-threonine phosphatase-interacting protein (PSTPIP)	Cell cycle control, cell division, chromosome partitioning
294963	0.77	1.95E-05	IPR007087 IPR015880	Zinc finger, C2H2-type, Zinc finger, C2H2-like	KOG4124	Putative transcriptional repressor regulating G2/M transition	Cell cycle control, cell division, chromosome partitioning
232886	0.73	3.06E-06	IPR001005 IPR009057	SANT, DNA-binding	KOG2043	Signaling protein SWIFT and related BRCT domain proteins	Cell cycle control, cell division, chromosome partitioning
232349	0.72	1.71E-03	IPR005045	Protein of unknown function DUF284	KOG2952	Cell cycle control protein	Signal transduction mechanisms
215598	-0.81	0.0016	IPR011025 IPR001019 IPR002975	G protein alpha subunit, helical insertion, Guanine nucleotide binding protein (G-protein), alpha subunit, Fungal G-protein, alpha subunit	KOG0082	G-protein alpha subunit (small G protein superfamily)	Cell cycle control, cell division, chromosome partitioning
222087	-1.27	7.48E-19	IPR000719 IPR017442	Protein kinase, core Serine/threonine protein kinase-related	KOG0590	Checkpoint kinase and related serine/threonine protein kinases	Cell cycle control, cell division, chromosome partitioning
