# Supplementary material for: Molecular Dialogues between Early Divergent Fungi and Bacteria in an Antagonism versus a Mutualism
Source: mBio. 2020 Sep 8;11(5):e02088-20. doi: 10.1128/mBio.02088-20 (PMC7482071; doi:10.1128/mBio.02088-20)
Supplement: FIG S1 [file mBio.02088-20-sf001.pdf]

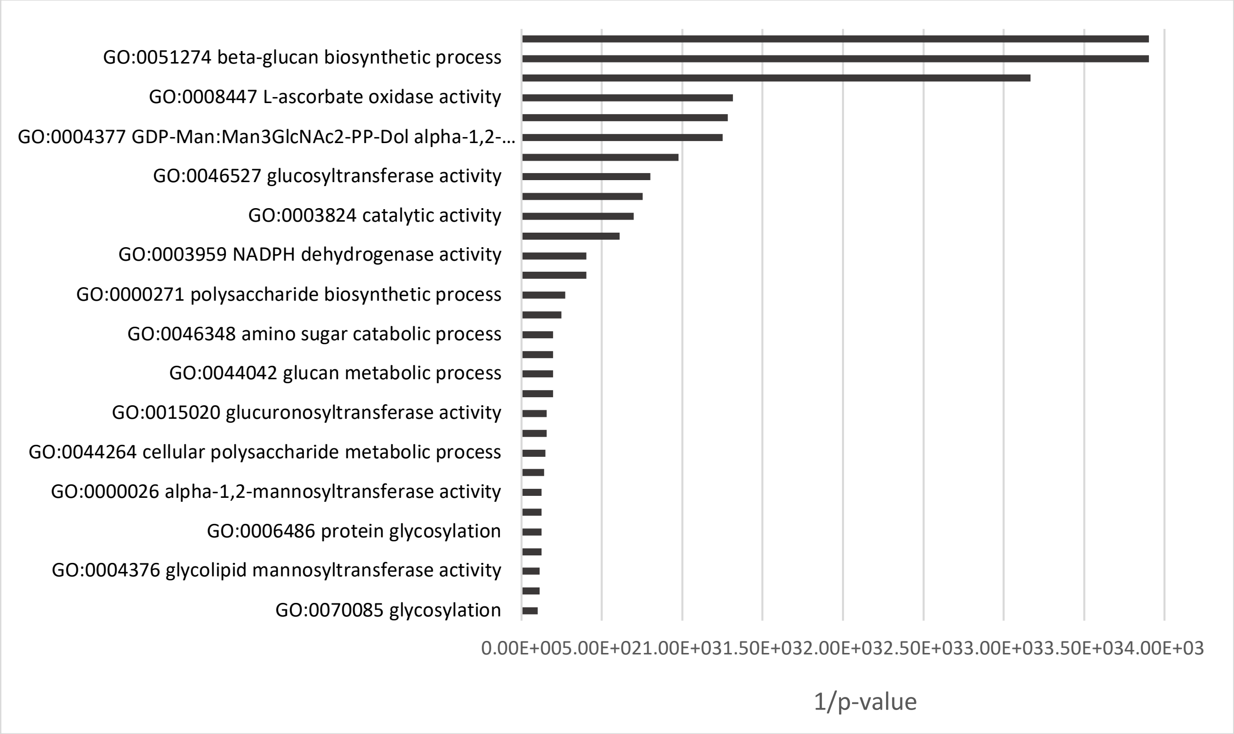

Figure S1. GO categories enriched in the upregulated fraction of the DE *Rm* non-host (ATCC 11559) genes in response to physical interaction with *Mecetohabitans* sp. B13.
