# Supplementary material for: Molecular Dialogues between Early Divergent Fungi and Bacteria in an Antagonism versus a Mutualism
Source: mBio. 2020 Sep 8;11(5):e02088-20. doi: 10.1128/mBio.02088-20 (PMC7482071; doi:10.1128/mBio.02088-20)
Supplement: TABLE S4 [file mBio.02088-20-st004.pdf]

Table S4. Candidate T3SS effectors upregulated in *Mycetohabitans* sp. B13 during pre-contact and physical contact with the *Rm* host (ATCC 52813) and the *Rm* non-host (ATCC 11559). ND, not detected.

Protein ID	Effective T3 Score	Eukaryotic-like domain	IMG annotation
**Pre-contact**			
2599762763	1	ND	hypothetical protein
2599763016	1	ND	hypothetical protein
2599763274	1	PF11937 10	Protein of unknown function (DUF3455)
2599763295	1	ND	hypothetical protein
2599763359	1	ND	hypothetical protein
2599763360	1	ND	hypothetical protein
2599763806	1	ND	hypothetical protein
2599764438	1	PF00158 5;PF02954 4	Response regulator containing CheY-like receiver, AAA-type ATPase, and DNA-binding domains
2599764562	1	ND	hypothetical protein
2599764578	1	ND	hypothetical protein
2599765017	1	ND	prevent-host-death family protein
2599765018	0.99955	ND	putative toxin-antitoxin system toxin component, PIN family
2599765428	0.99955	ND	hypothetical protein
2599765571	0.98923	PF01557 6	2-keto-4-pentenoate hydratase
2599765640	1	ND	Transglycosylase SLT domain
2599765794	1	PF00483 5;PF01050 5	mannose-1-phosphate guanylyltransferase/mannose-6-phosphate isomerase
**Physical contact**			
2599762710	1	ND	hypothetical protein
2599762745	1	ND	hypothetical protein
2599762753	1	ND	hypothetical protein
2599762755	1	PF00106 5	Dehydrogenases with different specificities (related to short-chain alcohol dehydrogenases)
2599762763	1	ND	hypothetical protein
2599763016	1	ND	hypothetical protein
2599763051	1	ND	hypothetical protein
2599763258	1	ND	hypothetical protein
2599763274	1	PF11937 10	Protein of unknown function (DUF3455)
2599763292	1	ND	hypothetical protein
2599763295	1	ND	hypothetical protein
2599763314	1	ND	hypothetical protein
2599763359	1	ND	hypothetical protein
2599763360	1	ND	hypothetical protein
2599763566	1	PF16576 4	Multidrug resistance efflux pump
2599763567	0.99997	PF07690 6	drug resistance transporter, EmrB/QacA subfamily
2599763623	1	PF13516 56	Leucine Rich repeat
2599763806	1	ND	hypothetical protein
2599763826	1	PF04632 7	Predicted membrane protein

Protein ID	Effective T3 Score	Eukaryotic-like domain	IMG annotation
2599763864	1	PF00848 14;PF00355 9	Phenylpropionate dioxygenase and related ring-hydroxylating dioxygenases, large terminal subunit
2599763909	1	PF00528 7	phosphate ABC transporter membrane protein 2, PhoT family (TC 3.A.1.7.1)
2599763968	1	ND	anti sigma-E protein, RseA
2599764195	1	PF00180 4	isocitrate dehydrogenase (NADP) (EC 1.1.1.42)
2599764211	1	PF01649 16	SSU ribosomal protein S20P
2599764253	1	PF01025 4	Molecular chaperone GrpE (heat shock protein)
2599764438	1	PF00158 5;PF02954 4	Response regulator containing CheY-like receiver, AAA-type ATPase, and DNA-binding domains
2599764562	1	ND	hypothetical protein
2599764578	1	ND	hypothetical protein
2599764584	1	ND	phenylacetate-CoA oxygenase, PaaG subunit
2599764628	1	PF13561 5	Dehydrogenases with different specificities (related to short-chain alcohol dehydrogenases)
2599764718	1	ND	hypothetical protein
2599765017	1	ND	prevent-host-death family protein
2599765167	1	PF02812 4	Glutamate dehydrogenase/leucine dehydrogenase
2599765172	1	ND	hypothetical protein
2599765247	0.99994	PF02629 7;PF00549 6	succinyl-CoA synthetase (ADP-forming) alpha subunit (EC 6.2.1.5)
2599765375	1	ND	hypothetical protein
2599765431	0.99989	ND	*btl*13-19
2599765433	0.99996	PF00271 5	Superfamily II DNA and RNA helicases
2599765537	1	PF03972 10	MmgE/PrpD family
2599765541	1	PF07702 5;PF00392 6	Transcriptional regulators
2599765547	1	PF00285 7	citrate synthase (EC 2.3.3.1)
2599765640	1	ND	Transglycosylase SLT domain
2599765719	1	ND	hypothetical protein
2599765780	1	PF12937 6	F-box-like
2599765782	0.99965	ND	hypothetical protein
2599765789	1	ND	O-antigen ligase like membrane protein
2599765794	1	PF00483 5;PF01050 5	mannose-1-phosphate guanylyltransferase/mannose-6-phosphate isomerase
2599765796	1	ND	hypothetical protein
