# Supplementary material for: Molecular Dialogues between Early Divergent Fungi and Bacteria in an Antagonism versus a Mutualism
Source: mBio. 2020 Sep 8;11(5):e02088-20. doi: 10.1128/mBio.02088-20 (PMC7482071; doi:10.1128/mBio.02088-20)
Supplement: TABLE S5 [file mBio.02088-20-st005.pdf]

Table S5. DE *Mycetohabitans* sp. B13 genes involved in ROS detoxification during physical interaction with host *Rm* ATCC 52813 and non-host *Rm* ATCC 11559 fungi. Log_2FC, log_2 fold change; positive Log_2FC values denote upregulated genes, negative Log_2FC values denote downregulated genes; FDR, false discovery rate.

Protein ID	Log_2FC	FDR	Annotation
**In response to non-host**			
2599764464	1.31422836	1.46E-07	Glutathione S-transferase
2599765053	1.05582174	6.05E-07	Glutathione synthase (EC 6.3.2.3)
2599763895	2.81256826	1.53E-46	Thioredoxin
2599765053	1.05582174	6.05E-07	Glutathione synthase (EC 6.3.2.3)
2599764015	1.29351515	0.00906441	Coenzyme A pyrophosphatase
2599764292	2.53968089	2.56e-17	Glutathione S-transferase
2599764121	-0.9111365	0.00245821	Cu/Zn superoxide dismutase
2599762957	-3.3502828	2.03E-10	Dyp-type peroxidase family
2599765091	-2.5725157	0.0055855	Glutathione S-transferase, C-terminal domain
**In response to host**			
2599764292	1.53715362	0.0085	Glutathione S-transferase
