# Supplementary material for: Molecular Dialogues between Early Divergent Fungi and Bacteria in an Antagonism versus a Mutualism
Source: mBio. 2020 Sep 8;11(5):e02088-20. doi: 10.1128/mBio.02088-20 (PMC7482071; doi:10.1128/mBio.02088-20)
Supplement: TABLE S6 [file mBio.02088-20-st006.pdf]

Table S6. *Mycetohabitans* and *Burkholderia* species and strains used in the OrthoMCL clustering analysis.

Organism	Source	Citation
Mycetohabitans rhizoxinica HKI 454	*Rm* ATCC 62417	(1)
Mycetohabitans sp. B13	*Rm* ATCC 52813	This study
Mycetohabitans sp. B14	*Rm* ATCC 52814	This study
B. ambifaria AMMD [LMG 19182]	Plant rhizosphere	JGI Genome Portal
B. cenocepacia AU1054	Blood of cystic fibrosis patient	JGI Genome Portal
B. cenocepacia J2315	Cystic fibrosis patient	(2)
Burkholderia sp. GG4	Ginger rhizosphere	(3)
B. dolosa AUO158	Causal agent of necrotizing pneumonia	(4)
B. glumae BGR1	Diseased rice pinacle	(5)
B. gladioli BSR3	Diseased rice sheath	(6)
B. mallei ATCC 23344	Agent of glanders	(7)
B. mallei NCTC 10247	Agent of glanders and pneumonia	(8)
B. multivorans ATCC 17616	Soil	(9)
B. phenoliruptrix BR3459a (CLA1)	Nitrogen-fixing symbiont of *Mimosa flocculosa*	(10)
B. phymatum STM815	Root nodule of *Machaerium lunatum*	(11)
B. phytofirmans PsJN T	Endophyte of onion roots	JGI Genome Portal
B. pseudomallei BPC006	Melioidosis patient	(12)
B. thailandensis E264	Non-pathogenic saprophyte	(13)
B. vietnamiensis G4	Wastewater treatment facility	JGI Genome Portal
B. xenovorans LB400 (formerly *B. fungorum*)	Landfill	JGI Genome Portal

References

1. Lackner G, Moebius N, Partida-Martinez L, Hertweck C. 2011. Complete genome sequence of *Burkholderia rhizoxinica*, an endosymbiont of *Rhizopus microsporus*. Journal of Bacteriology 193:783-784.
2. Holden MT, Seth-Smith HM, Crossman LC, Sebaihia M, Bentley SD, Cerdeno-Tarraga AM, Thomson NR, Bason N, Quail MA, Sharp S, Cherevach I, Churcher C, Goodhead I, Hauser H, Holroyd N, Mungall K, Scott P, Walker D, White B, Rose H, Iversen P, Mil-Homens D, Rocha EP, Fialho AM, Baldwin A, Dowson C, Barrell BG, Govan JR, Vandamme P, Hart CA, Mahenthiralingam E, Parkhill J. 2009. The genome of *Burkholderia cenocepacia* J2315, an epidemic pathogen of cystic fibrosis patients. Journal of Bacteriology 191:261-277.
3. Hong KW, Koh CL, Sam CK, Yin WF, Chan KG. 2012. Complete genome sequence of *Burkholderia* sp. Strain GG4, a betaproteobacterium that reduces 3-oxo-N-acylhomoserine lactones and produces different N-acylhomoserine lactones. Journal of Bacteriology 194:6317.
4. Johnson SL, Bishop-Lilly KA, Ladner JT, Daligault HE, Davenport KW, Jaissle J, Frey KG, Koroleva GI, Bruce DC, Coyne SR, Broomall SM, Li PE, Teshima H, Gibbons HS, Palacios GF, Rosenzweig CN, Redden CL, Xu Y, Minogue TD, Chain PS. 2015. Complete genome sequences for 59 *Burkholderia* isolates, both pathogenic and near neighbor. Genome Announcements 3:e00159-15.
5. Lim J, Lee TH, Nahm BH, Choi YD, Kim M, Hwang I. 2009. Complete genome sequence of *Burkholderia glumae* BGR1. Journal of Bacteriology 191:3758-3759.
6. Seo YS, Lim J, Choi BS, Kim H, Goo E, Lee B, Lim JS, Choi IY, Moon JS, Kim J, Hwang I. 2011. Complete genome sequence of *Burkholderia gladioli* BSR3. Journal of Bacteriology 193:3149.

7. Nierman WC, DeShazer D, Kim HS, Tettelin H, Nelson KE, Feldblyum T, Ulrich RL, Ronning CM, Brinkac LM, Daugherty SC, Davidsen TD, Deboy RT, Dimitrov G, Dodson RJ, Durkin AS, Gwinn ML, Haft DH, Khouri H, Kolonay JF, Madupu R, Mohammoud Y, Nelson WC, Radune D, Romero CM, Sarria S, Selengut J, Shamblin C, Sullivan SA, White O, Yu Y, Zafar N, Zhou L, Fraser CM. 2004. Structural flexibility in the *Burkholderia mallei* genome. Proceedings of the National Academy of Sciences of the United States of America 101:14246-14251.
8. Daligault HE, Davenport KW, Minogue TD, Bishop-Lilly KA, Broomall SM, Bruce DC, Chain PS, Coyne SR, Frey KG, Gibbons HS, Jaissle J, Koroleva GI, Ladner JT, Lo CC, Munk C, Palacios GF, Redden CL, Rosenzweig CN, Scholz MB, Johnson SL. 2014. Whole-genome assemblies of 56 *Burkholderia* species. Genome Announcements 2:e01106-14.
9. Nishiyama E, Ohtsubo Y, Nagata Y, Tsuda M. 2010. Identification of *Burkholderia multivorans* ATCC 17616 genes induced in soil environment by in vivo expression technology. Environmental Microbiology 12:2539-2558.
10. de Oliveira Cunha C, Goda Zuleta LF, Paula de Almeida LG, Prioli Ciapina L, Lustrino Borges W, Pitard RM, Baldani JI, Straliotto R, de Faria SM, Hungria M, Sousa Cavada B, Mercante FM, Ribeiro de Vasconcelos AT. 2012. Complete genome sequence of *Burkholderia phenoliruptrix* BR3459a (CLA1), a heat-tolerant, nitrogen-fixing symbiont of *Mimosa flocculosa*. Journal of Bacteriology 194:6675-6676.
11. Moulin L, Klonowska A, Caroline B, Booth K, Vriezen JA, Melkonian R, James EK, Young JP, Bena G, Hauser L, Land M, Kyrpides N, Bruce D, Chain P, Copeland A, Pitluck S, Woyke T, Lizotte-Waniewski M, Bristow J, Riley M. 2014. Complete Genome sequence of *Burkholderia phymatum* STM815(T), a broad host range and efficient nitrogen-fixing symbiont of *Mimosa* species. Standards in Genomic Sciences 9:763-774.
12. Fang Y, Huang Y, Li Q, Chen H, Yao Z, Pan J, Gu J, Tang B, Wang HG, Yu B, Tong YG, Zou QM, Mao XH. 2012. First genome sequence of a *Burkholderia pseudomallei* isolate in China, strain BPC006, obtained from a melioidosis patient in Hainan. Journal of Bacteriology 194:6604-6605.
13. Kim HS, Schell MA, Yu Y, Ulrich RL, Sarria SH, Nierman WC, DeShazer D. 2005. Bacterial genome adaptation to niches: divergence of the potential virulence genes in three *Burkholderia* species of different survival strategies. BMC Genomics 6:174.
