# Supplementary material for: Molecular Dialogues between Early Divergent Fungi and Bacteria in an Antagonism versus a Mutualism
Source: mBio. 2020 Sep 8;11(5):e02088-20. doi: 10.1128/mBio.02088-20 (PMC7482071; doi:10.1128/mBio.02088-20)
Supplement: TABLE S7 [file mBio.02088-20-st007.pdf]

Table S7. *Mycetohabitans* sp. B13 genes encoded exclusively in genomes of endofungal bacteria and upregulated during physical contact with host and non-host fungi. Annotations provided by JGI Integrated Microbial Genomes and Microbiomes. T3SS signals are predicted by Effective T3 V2.0 (1).

Protein ID	Annotation	T3SS signal
2599762710	Hypothetical protein	Present
2599762721	Hypothetical protein	Absent
2599762734	Acetyltransferase (GNAT) family	Absent
2599762742	Hypothetical protein	Present
2599762754	Glycosyl hydrolase catalytic core	Absent
2599762763	Hypothetical protein	Present
2599762840	Acyl carrier protein	Absent
2599762841	Acyl-CoA dehydrogenases	Absent
2599762848	Response regulators consisting of a CheY-like receiver domain and a winged-helix DNA-binding domain	Present
2599762998	Glycine zipper	Absent
2599763016	Hypothetical protein	Present
2599763051	Hypothetical protein	Present
2599763251	Methionyl-tRNA formyltransferase	Absent
2599763268	Bacterial type III secretion protein (HrpB1_HrpK)	Absent
2599763281	Bacterial type III secretion protein (HrpB2)	Absent
2599763289	Hypothetical protein	Absent
2599763292	Hypothetical protein	Present
2599763295	Hypothetical protein	Present
2599763360	Hypothetical protein	Present
2599763422	Hypothetical protein	Absent
2599763623	Leucine Rich repeat	Present
2599763727	Conserved hypothetical phage tail region protein	Absent
2599764053	Hypothetical protein	Absent
2599764576	Hypothetical protein	Absent
2599764986	Hypothetical protein	Absent
2599765016	Hypothetical protein	Absent
2599765415	Hypothetical protein	Absent
2599765428	Hypothetical protein	Present
2599765431	*Xanthomonas* avirulence protein, Avr/PthA	Present
2599765635	Hypothetical protein	Absent
2599765640	Transglycosylase SLT domain	Present
2599765719	Hypothetical protein	Present
2599765780	F-box-like	Present
2599765789	O-antigen ligase like membrane protein	Present
2599765858	Transposase domain (DUF772)/Transposase DDE domain	Absent

1. Jehl MA, Arnold R, Rattei T. 2011. Effective—a database of predicted secreted bacterial proteins. Nucleic Acids Research 39:D591-D595.
