# Supplementary material for: Molecular Dialogues between Early Divergent Fungi and Bacteria in an Antagonism versus a Mutualism
Source: mBio. 2020 Sep 8;11(5):e02088-20. doi: 10.1128/mBio.02088-20 (PMC7482071; doi:10.1128/mBio.02088-20)
Supplement: TABLE S8 [file mBio.02088-20-st008.pdf]

Table S8. Mucoromycotina isolates screened for the presence of *Burkholderia*-related endobacteria.

Fungal isolate	**Endobacteria**
Absidia glauca CU 84-173	Absent
Cokeromyces recurvatus CU 95-158	Absent
Cunninghamella blakesleeana CU 95-022	Absent
Cunninghamella echinulata CU 92-173	Absent
Gongronella butleri CU 84-094	Absent
Mucor circinelloides CU 92-040	Absent
Mucor circinelloides CBS 277.49	Absent
Mucor genevensis CU 84-174	Absent
Mucor hiemalis CU 91-143	Absent
Mucor racemosus CU 93-008	Absent
Mycotypha africana CU 92-094	Absent
Phycomyces blakesleeanus CU 98-005	Absent
Radiomyces embreei CU 93-126	Absent
Rhizopus arrhizus NRRL 1469	Absent
Rhizopus arrhizus NRRL 2582	Absent
Rhizopus arrhizus NRRL 54538	Absent
Rhizopus arrhizus NRRL 54539	Absent
Rhizopus azygosporus DUKE 8560	Absent
Rhizopus delemar ATCC 36412	Absent
Rhizopus delemar CNRMA 03.253	Absent
Rhizopus delemar CNRMA 04.160	Absent
Rhizopus delemar CBS 389.34	Absent
Rhizopus delemar FSU 6025	Absent
Rhizopus delemar NRRL A-21447	Absent
Rhizopus delemar NRRL 1472	Absent
Rhizopus delemar NRRL 1705	Absent
Rhizopus delemar NRRL 3562	Absent
Rhizopus delemar NRRL 3563	Absent
Rhizopus homothallicus DUKE 7693	Absent
Rhizopus microsporus NRRL 5905	Absent
Rhizopus microsporus NRRL 54029	Absent
Rhizopus microsporus ATCC 11559	Absent
Rhizopus microsporus ATCC 52807	Absent
Rhizopus microsporus ATCC 56018	Absent
Rhizopus microsporus ATCC 20577	**Present**
Rhizopus microsporus ATCC 46348	**Present**
Rhizopus microsporus ATCC 52811	**Present**
Rhizopus microsporus ATCC 52812	**Present**
Rhizopus microsporus ATCC 52813	**Present**

Fungal isolate	**Endobacteria**
Rhizopus microsporus ATCC 52814	**Present**
Rhizopus microsporus ATCC 56019	**Present**
Rhizopus microsporus ATCC 56028	**Present**
Rhizopus microsporus ATCC 62417	**Present**
Rhizopus oryzae ATCC 11423	Absent
Rhizopus oryzae ATCC 13440	Absent
Rhizopus oryzae ATCC 44170	Absent
Rhizopus oryzae ATCC 56536	Absent
Rhizopus oryzae FSU 5858	Absent
Rhizopus oryzae FSU 6020	Absent
Rhizopus oryzae FSU 6024	Absent
Rhizopus oryzae NRRL 395	Absent
Rhizopus oryzae NRRL 1526	Absent
Rhizopus oryzae NRRL 2871	Absent
Rhizopus oryzae NRRL 2908	Absent
Rhizopus oryzae NRRL 6431	Absent
Rhizopus oryzae NRRL A-21789	Absent
Rhizopus oryzae CU 99-892	Absent
Rhizopus sexualis DUKE 7694	Absent
Rhizopus stolonifer CU 92-208	Absent
Rhizopus stolonifer 92-RS-5	Absent
Syncephalastrum racemosum CU 91-132	Absent
Thamnidium elegans CU 95-083	Absent
Umbelopsis versiformis CU 91-049	Absent
Zygorhynchus heterogamous CU 92-287	Absent
