# Supplementary material for: Molecular Dialogues between Early Divergent Fungi and Bacteria in an Antagonism versus a Mutualism
Source: mBio. 2020 Sep 8;11(5):e02088-20. doi: 10.1128/mBio.02088-20 (PMC7482071; doi:10.1128/mBio.02088-20)
Supplement: TABLE S9 [file mBio.02088-20-st009.pdf]

Table S9. Secondary metabolite gene (SMG) clusters in genomes of Eurotiomycetes (Ascomycota) and Mucoromycotina. Data obtained from the JGI Mycocosm website (https://mycocosm.jgi.doe.gov/mycocosm/home), accessed on 3 June 2020. DMAT, dimethylallyl transferase; NRPS, nonribosomal peptide synthetase; PKS, polyketide synthase; TC, terpene cyclase.

Genome	DMAT	HYBRID	NRPS	NRPS-Like	PKS	PKS-Like	TC	Total
**EUROTIOMYCETES**								
Arthroderma benhamiae CBS 112371	2	3	14	4	7	2	2	34
Aspergillus acristatulus CBS 119.55 v1.0	3	2	12	14	31	3	0	65
Aspergillus aculeatinus CBS 121060 v1.0	6	2	19	20	28	3	3	81
Aspergillus aculeatus ATCC16872 v1.1	3	4	18	21	19	4	2	71
Aspergillus affinis CBS 129190 v1.0	13	8	13	22	36	3	6	101
Aspergillus alabamensis v1.0	7	4	14	16	21	3	3	68
Aspergillus albertensis v1.0	5	2	13	17	38	7	4	86
Aspergillus allahabadii v1.0	10	3	10	19	16	2	1	61
Aspergillus alliaceus CBS 536.65 v1.0	2	2	15	20	37	8	5	89
Aspergillus ambiguus v1.0	8	1	11	18	18	1	2	59
Aspergillus amoeneus CBS 111.32 v1.0	10	3	14	12	19	4	0	62
Aspergillus amylovorus CBS 600.67 v1.0	2	3	11	9	27	2	6	60
Aspergillus arachidicola v1.0	10	3	18	13	18	5	3	70
Aspergillus arxii CBS 52583 v1.0	1	1	4	10	11	2	0	29
Aspergillus assulatus CBS 27911 v1.0	2	1	4	7	9	2	4	29
Aspergillus astellatus v1.0	3	6	12	15	29	6	5	76
Aspergillus aurantiobrunneus CBS 465.65 v1.0	6	1	10	14	20	3	6	60
Aspergillus aureofulgens IBT 27100 v1.0	11	0	12	18	13	2	1	57
Aspergillus aureolatus CBS 190.65 v1.0	7	3	15	6	36	5	3	75
Aspergillus aureoluteus CBS 105.55 v1.0	5	2	6	8	9	3	3	36
Aspergillus aureoterreus v1.0	8	2	14	14	17	3	6	64
Aspergillus avenaceus IBT 18842 v1.0	1	4	11	18	18	4	6	62
Aspergillus bertholletius IBT 29228 v1.0	6	5	11	10	22	5	6	65
Aspergillus biplanus CBS 468.65 v1.0	5	1	11	19	34	6	7	83
Aspergillus bisporus CBS 707.71 v1.0	2	1	8	12	12	4	2	41
Aspergillus bombycis NRRL 26010	7	4	18	18	22	9	5	83
Aspergillus botucatensis CBS 114221 v1.0	10	2	14	8	16	4	1	55
Aspergillus brasiliensis v1.0	2	5	11	17	29	6	3	73
Aspergillus brevijanus CBS 111.46 v1.0	10	3	20	23	39	3	4	102
Aspergillus brevipes CBS 118.53 v1.0	4	3	8	18	19	3	2	57
Aspergillus brunneoviolaceus CBS 621.78 v1.0	4	2	21	21	24	3	4	79
Aspergillus caelatus CBS 763.97 v1.0	8	4	16	20	22	8	5	83
Aspergillus calidoustus	7	1	9	13	28	2	6	66

Genome	DMAT	HYBRID	NRPS	NRPS-Like	PKS	PKS-Like	TC	Total
Aspergillus californicus CBS 123895 v1.0	8	5	19	16	29	4	6	87
Aspergillus campestris IBT 28561 v1.0	5	5	11	11	12	2	2	48
Aspergillus candidus CBS 102.13 v1.0	4	4	11	6	16	3	1	45
Aspergillus caninus CBS 128032 v1.0	0	0	3	3	9	2	0	17
Aspergillus capensis CBS 138188 v1.0	6	3	20	18	13	3	5	68
Aspergillus carbonarius ITEM 5010 v3	2	5	11	20	18	4	4	64
Aspergillus carlsbadensis CBS 123894 v1.0	5	2	9	16	26	3	7	68
Aspergillus cervinus CBS 196.64 v1.0	3	1	16	10	24	5	4	63
Aspergillus chevalieri CBS 522.65 v1.0	2	1	3	10	13	2	0	31
Aspergillus chrysellus CBS 472.65 v1.0	7	11	14	26	38	5	4	105
Aspergillus clavatonanicus v1.0	4	4	11	7	15	3	3	47
Aspergillus clavatus NRRL 1 from AspGD	3	4	10	5	16	3	4	45
Aspergillus cleistominutus CBS 200.75 v1.0	6	2	15	14	27	2	3	69
Aspergillus coreanus CBS 117059 v1.0	8	1	11	10	14	2	1	47
Aspergillus coremiiformis CBS 553.77 v1.0	5	0	6	8	15	2	2	38
Aspergillus costaricaensis CBS 115574 v1.0	3	5	17	21	32	7	7	92
Aspergillus costiformis CBS 101749 v1.0	2	0	5	10	11	2	0	30
Aspergillus creber IBT 32277 v1.0	9	4	17	11	21	4	1	67
Aspergillus cretensis CBS 112802 v1.0	12	10	14	21	31	6	4	98
Aspergillus cristatus GZAAS20.1005	1	0	5	13	11	2	1	33
Aspergillus crustosus CBS 478.65 v1.0	7	0	7	11	18	3	3	49
Aspergillus cumulatus DTO 311-F5 v1.0	2	0	4	9	9	4	0	28
Aspergillus dentatus CBS 114.63 v1.0	6	2	10	16	24	5	1	64
Aspergillus desertorum CBS 653.73 v1.0	4	3	7	12	20	9	5	60
Aspergillus dromiae CBS 140633 v1.0	5	2	8	14	35	3	6	73
Aspergillus duricaulis CBS 481.65 v1.0	7	2	4	10	24	2	4	53
Aspergillus egyptiacus CBS 656.73 v1.0	4	0	16	8	19	4	4	55
Aspergillus elegans CBS 116.39 v1.0	8	8	17	26	27	3	5	94
Aspergillus ellipticus CBS 707.79 v1.0	2	2	15	14	28	7	4	72
Aspergillus eucalypticola CBS 122712 v1.0	2	6	11	19	26	4	6	74
Aspergillus falconensis CBS 271.91 v1.0	5	2	12	13	33	5	2	72
Aspergillus ferenczii CBS 121594 v1.0	2	2	7	9	12	2	3	37
Aspergillus fijiensis CBS 313.89 v1.0	5	3	21	25	26	3	4	87
Aspergillus filifera CBS 114510 v1.0	5	1	10	13	30	3	3	65
Aspergillus flavipes CBS 22552 v1.0	7	3	19	26	28	6	3	92
Aspergillus flavus NRRL3357	10	1	16	16	20	7	3	73
Aspergillus floccosus CBS 116.37 v1.0	10	5	19	23	26	3	3	89

Genome	DMAT	HYBRID	NRPS	NRPS-Like	PKS	PKS-Like	TC	Total
Aspergillus flocculosus v1.0	8	5	18	24	28	2	4	89
Aspergillus floridensis DTO 198-A8 v1.0	4	1	16	21	23	4	3	72
Aspergillus foveolata CBS 279.81 v1.0	5	2	8	17	29	4	3	68
Aspergillus frequens CBS 586.65 v1.0	8	2	13	24	16	2	4	69
Aspergillus fructiculosus v1.0	5	2	10	14	38	2	2	73
Aspergillus fumigatiaffinis CBS 117186 v1.0	7	2	19	12	23	3	3	69
Aspergillus fumigatus A1163	3	2	8	6	11	2	0	32
Aspergillus fumigatus Af293 from AspGD	2	0	9	7	14	2	0	34
Aspergillus fumisynnematus IBT 28474 v1.0	8	2	13	10	20	4	2	59
Aspergillus funiculosus CBS 116.56 v1.0	5	0	5	5	15	2	3	35
Aspergillus galapagensis CBS 117522 v1.0	3	2	9	9	12	2	2	39
Aspergillus germanicus CBS 123887 v1.0	5	0	10	16	24	3	7	65
Aspergillus giganteus CBS 515.65 v1.0	3	3	12	7	18	3	2	48
Aspergillus glaucus v1.0	1	0	6	11	13	3	0	34
Aspergillus granulosus CBS 588.65 v1.0	8	0	8	13	22	2	5	58
Aspergillus haitiensis CBS 468.91 v1.0	3	0	6	10	29	3	8	59
Aspergillus heteromorphus CBS 117.55 v1.0	4	3	12	16	16	3	3	57
Aspergillus heterothallicus CBS 489.65 v1.0	4	0	9	16	15	4	4	52
Aspergillus hiratsukae CBS 294.93 v1.0	4	2	10	9	10	3	2	40
Aspergillus homomorphus CBS 101889 v1.0	4	3	16	22	24	5	4	78
Aspergillus ibericus CBS 121593 v1.0	2	6	7	18	15	6	3	57
Aspergillus igneus CBS 466.65 v1.0	5	4	13	10	23	2	1	58
Aspergillus iizukae CBS 541.69 v1.0	7	4	18	22	16	3	3	73
Aspergillus implicatus CBS 484.95 v1.0	3	4	13	18	49	5	8	100
Aspergillus indicus v2.0	7	2	10	14	29	2	2	66
Aspergillus indologenus CBS 114.80 v1.0	6	5	17	24	27	3	2	84
Aspergillus insolitus CBS 384.61 v1.0	0	1	9	7	9	2	1	29
Aspergillus insuetus CBS 107.25 v1.0	5	1	8	17	27	2	6	66
Aspergillus japonicus CBS 114.51 v1.0	4	1	21	22	19	3	1	71
Aspergillus karnatakaensis CBS 102800 v1.0	11	3	12	19	29	5	2	81
Aspergillus kawachii IFO 4308	2	6	15	17	33	5	6	84
Aspergillus keveii CBS 209.92 v1.0	8	0	11	17	25	3	8	72
Aspergillus laciniosus CBS 117721 v1.0	9	2	16	11	15	5	1	59
Aspergillus lentulus CBS 117885 v1.0	6	2	12	9	18	3	2	52
Aspergillus leporis CBS 151.66 v1.0	8	6	17	23	25	4	3	86
Aspergillus longivesica v1.0	3	4	12	8	10	5	2	44

Genome	DMAT	HYBRID	NRPS	NRPS-Like	PKS	PKS-Like	TC	Total
Aspergillus luchuensis CBS 106.47 v1.0	2	6	16	22	30	5	6	87
Aspergillus lucknowensis CBS 449.75 v1.0	3	3	7	6	23	5	3	50
Aspergillus luppii CBS 653.74 v1.0	10	0	14	19	12	3	1	59
Aspergillus microcysticus CBS 120.58 v1.0	0	2	8	14	13	2	1	40
Aspergillus minisclerotigenes CBS 117635 v1.0	11	1	16	18	26	7	4	83
Aspergillus miraensis CBS 140625 v1.0	5	3	7	13	34	4	4	70
Aspergillus multicolor v1.0	4	3	10	17	28	5	2	69
Aspergillus multiplicatus CBS 646958 v1.0	2	1	4	8	6	3	3	27
Aspergillus muricatus v1.0	8	3	14	22	29	3	4	83
Aspergillus nakazawae v1.0	9	3	10	25	33	3	7	90
Aspergillus navahoensis v1.0	4	2	11	17	33	5	2	74
Aspergillus neoauricomus CBS112787 v1.0	6	5	10	23	33	3	4	84
Aspergillus neoconicus CBS 233.90 v1.0	1	0	7	8	8	3	0	27
Aspergillus neoechinulatus CBS120.55 v1.0	4	0	9	11	28	3	2	57
Aspergillus neoflavipes CBS 260.73 v1.0	9	3	19	25	18	2	3	79
Aspergillus neoindicus CBS 444.75 v1.0	8	2	17	19	20	3	2	71
Aspergillus neoniger CBS 115656 v1.0	3	4	13	16	29	6	9	80
Aspergillus neowarcupii IBT 29024 v1.0	3	1	10	13	21	2	3	53
Aspergillus nidulans	5	1	9	13	22	5	1	56
Aspergillus niger (lacticoffeatus) CBS 101883 v1.0	2	9	16	16	23	6	7	79
Aspergillus niger (phoenicis Corda) Thom ATCC 13157 v1.0	2	9	15	18	26	5	6	81
Aspergillus niger ATCC 1015 v4.0	2	9	16	16	27	4	6	80
Aspergillus niger CBS 513.88	2	6	14	15	29	5	6	77
Aspergillus niger NRRL3	2	9	16	16	29	4	6	82
Aspergillus niger van Tieghem ATCC 13496 v1.0	2	8	15	14	29	4	7	79
Aspergillus nishimurae IFM 54133 v1.0	2	1	2	9	6	3	3	26
Aspergillus nomius IBT 12657 v1.0	7	1	16	18	24	6	3	75
Aspergillus nomius NRRL 13137	5	3	19	19	26	5	4	81
Aspergillus novofumigatus IBT 16806 v1.0	4	3	16	5	24	2	2	56
Aspergillus novoparasiticus CBS 126849 v1.0	8	1	16	18	25	6	3	77
Aspergillus nutans CBS 121.56 v1.0	3	2	27	15	15	4	2	68
Aspergillus ochraceoroseus IBT 24754 v1.0	4	2	6	3	10	2	1	28
Aspergillus ochraceoroseus SRRC1432	4	1	6	4	9	3	1	28
Aspergillus oerlinghausenensis CBS 139183 v1.0	7	0	10	7	12	2	0	38
Aspergillus olivicola v1.0	5	2	7	12	29	4	3	62

Genome	DMAT	HYBRID	NRPS	NRPS-Like	PKS	PKS-Like	TC	Total
Aspergillus oryzae RIB40	9	2	14	15	23	5	2	70
Aspergillus ostianus v1.0	8	4	12	17	34	4	4	83
Aspergillus paleaceus CBS 498.65 v1.0	2	0	7	7	8	3	1	28
Aspergillus papuensis CBS 841.96 v1.0	4	2	6	10	10	1	3	36
Aspergillus parasiticus CBS 117618 v1.0	11	0	22	17	26	6	5	87
Aspergillus parvisclerotigenus CBS 121.62 v1.0	8	4	14	14	20	5	3	68
Aspergillus parvulus CBS 136.61 v1.0	5	2	18	14	18	6	1	64
Aspergillus penicilloides CBS 540.65 v1.0	1	1	6	6	11	3	0	28
Aspergillus pernambucoensis CBS 137449 v1.0	5	0	7	10	6	2	2	32
Aspergillus persii CBS 112795 v1.0	6	10	16	24	43	4	3	106
Aspergillus petrakii CBS 105.57 v1.0	7	4	13	20	35	3	4	86
Aspergillus piperis CBS 112811 v1.0	2	6	14	20	30	5	8	85
Aspergillus primulinus CBS 253.94 v1.0	2	0	6	9	7	3	1	28
Aspergillus pseudocaelatus CBS 117616 v1.0	8	3	15	18	18	10	4	76
Aspergillus pseudodeflectus v1.0	5	1	9	15	26	4	6	66
Aspergillus pseudofelis IBT 34107 v1.0	7	3	19	11	30	2	3	75
Aspergillus pseudonomius CBS 119388 v1.0	6	1	17	19	23	6	3	75
Aspergillus pseudotamarii CBS 117625 v1.0	7	5	16	19	24	7	4	82
Aspergillus pseudoterreus DTO 47-E6 v1.0	8	5	11	16	23	2	2	67
Aspergillus pseudoustus CBS 123904 v1.0	6	0	15	14	26	3	4	68
Aspergillus pseudoviridinutans IBT 34175 v1.0	7	1	18	13	28	5	2	74
Aspergillus pulvericola CBS 137327 v1.0	10	6	13	25	34	3	4	95
Aspergillus puulaauensis IBT 32284 v1.0	9	3	12	15	22	3	1	65
Aspergillus quadrilineatus (floriformis) CBS 937.73 v1.0	3	2	9	13	34	8	2	71
Aspergillus quadrilineatus (gemmatus) CBS 853.96 v1.0	3	3	11	16	29	6	3	71
Aspergillus rambellii 100887 v1.0	3	2	6	4	11	2	1	29
Aspergillus rambellii SRRC1468	3	1	8	4	11	3	1	31
Aspergillus recurvatus v1.0	5	2	8	12	31	6	2	66
Aspergillus restrictus CBS 118.33 v1.0	0	1	4	9	6	2	0	22
Aspergillus rhizopodus v1.0	4	4	14	6	18	4	3	53
Aspergillus robustus CBS 428.77 v1.0	2	4	20	16	19	4	5	70
Aspergillus roseoglobulosus CBS112800 v1.0	8	6	16	27	34	4	8	103
Aspergillus saccharolyticus JOP 1030-1 v1.0	3	2	14	15	11	6	0	51
Aspergillus sclerotiicarbonarius CBS 121057 v1.0	3	6	14	22	25	6	3	79

Genome	DMAT	HYBRID	NRPS	NRPS-Like	PKS	PKS-Like	TC	Total
Aspergillus sclerotioniger CBS115572 v1.0	3	5	9	21	21	5	5	69
Aspergillus sclerotiorum CBS 549.65 v1.0	6	11	14	24	38	4	4	101
Aspergillus sepultus CBS 257.85 v1.0	0	2	12	16	27	3	4	64
Aspergillus sergii CBS 130017 v1.0	9	0	16	22	26	7	3	83
Aspergillus sesamicola CBS 137324 v1.0	8	3	13	23	31	4	2	84
Aspergillus shendaweii IBT 34197 v1.0	4	0	7	8	8	4	4	35
Aspergillus siamensis CBS 137452 v1.0	4	1	17	11	19	4	2	58
Aspergillus silvaticus CBS 128.55 v1.0	6	4	8	10	21	5	2	56
Aspergillus similis v1.0	5	3	8	15	22	6	2	61
Aspergillus spathulatus CBS 408.89 v1.0	3	1	2	10	6	2	3	27
Aspergillus spectabilis CBS 429.77A v1.0	6	1	13	20	36	4	3	83
Aspergillus spinosus CBS 483.65 v1.0	10	2	12	8	14	4	2	52
Aspergillus stella-maris CBS 113639 v1.0	5	1	10	13	36	3	5	73
Aspergillus stercoraria CBS 428.93 v1.0	4	1	7	11	26	4	4	57
Aspergillus steynii IBT 23096 v1.0	9	9	17	22	29	3	4	93
Aspergillus subauricomus CBS 638.78 v1.0	7	4	18	20	34	3	7	93
Aspergillus sublatus IBT 19356 v1.0	4	1	10	13	28	4	3	63
Aspergillus subramanianii CBS 138230 v1.0	6	10	14	22	48	4	5	109
Aspergillus sulphureus CBS 550.65 v1.0	8	9	17	23	36	4	6	103
Aspergillus sydowii CBS 593.65 v1.0	7	2	13	11	17	2	0	52
Aspergillus taichungensis IBT 19404 v1.0	5	3	10	9	11	4	1	43
Aspergillus tamarii CBS 117626 v1.0	11	3	19	18	22	6	2	81
Aspergillus tanneri DTO 303-18 v1.0	7	9	23	12	41	5	4	101
Aspergillus templicola CBS 138181 v1.0	9	2	17	21	20	5	2	76
Aspergillus tennesseensis IBT 32283 v1.0	7	2	10	13	22	4	1	59
Aspergillus terreus NIH 2624	5	1	15	17	24	2	2	66
Aspergillus tetrazonus CBS 591.65A v1.0	4	1	11	14	28	7	2	67
Aspergillus thermomutatus v1.0	4	4	9	10	19	3	3	52
Aspergillus thesauricus IBT 34227 v1.0	6	1	7	15	21	2	6	58
Aspergillus transcarpathicus CBS 423.68 v1.0	4	0	19	10	17	5	5	60
Aspergillus transmontanensis CBS 130015 v1.0	12	2	14	16	28	5	3	80
Aspergillus trinidadensis IBT 32571 v1.0	5	1	17	25	22	3	1	74
Aspergillus triticus CBS266.81 v1.0	5	2	11	7	23	2	2	52
Aspergillus tsurutae IBT 34206 v1.0	3	0	7	7	10	3	1	31
Aspergillus tubingensis v1.0	2	7	14	19	31	6	6	85

Genome	DMAT	HYBRID	NRPS	NRPS-Like	PKS	PKS-Like	TC	Total
Aspergillus turcosus IBT 27921 v1.0	3	2	11	11	12	3	2	44
Aspergillus udagawae IFM 46973	5	3	13	10	25	3	3	62
Aspergillus undulatus CBS 261.88 v1.0	5	1	6	15	21	3	3	54
Aspergillus unguis CBS132.55 v1.0	1	2	9	15	13	2	1	43
Aspergillus uvarum CBS 121591 v1.0	4	3	17	21	21	3	2	71
Aspergillus vadensis CBS 113365 v1.0	2	5	13	22	27	5	4	78
Aspergillus varians CBS 505.65 v1.0	9	1	11	10	17	3	2	53
Aspergillus venezuelensis CBS 868.97 v1.0	7	1	13	12	37	4	3	77
Aspergillus versicolor v1.0	6	2	15	13	22	4	1	63
Aspergillus violaceofuscus CBS 115571 v1.0	6	3	19	20	17	4	3	72
Aspergillus viridinutans CBS 127.56 v1.0	6	2	10	9	21	3	5	56
Aspergillus waksmanii IBT 31900 v1.0	2	2	8	8	11	2	3	36
Aspergillus welwitschiae CBS139.54b v1.0	2	7	15	20	26	4	7	81
Aspergillus wentii v1.0	2	3	12	14	20	2	5	58
Aspergillus westerdijkiae CBS 112803 v1.0	10	5	16	22	30	5	6	94
Aspergillus westlandensis CBS 123905 v1.0	7	5	12	24	27	4	7	86
Aspergillus zonatus v1.0	1	0	6	8	12	3	2	32
Blastomyces dermatitidis SLH14081	2	0	4	4	1	2	0	13
Byssochlamys spectabilis No. 5	2	1	3	10	5	2	3	26
Caliciopsis orientalis	0	1	6	10	11	3	0	31
Capronia coronata CBS 617.96	0	0	3	3	2	3	0	11
Capronia epimyces CBS 606.96	0	0	2	7	6	2	0	17
Capronia fungicola CBS 614.96 v1.0	0	0	3	6	4	2	0	15
Capronia semiimmersa CBS27337	0	0	4	5	3	2	0	14
Cladophialophora bantiana CBS 173.52	1	0	2	8	2	3	0	16
Cladophialophora carrionii CBS 160.54	0	0	3	4	4	2	1	14
Cladophialophora immunda CBS83496	0	0	2	6	5	2	0	15
Cladophialophora psammophila CBS 110553	0	0	2	10	2	2	0	16
Cladophialophora yegresii CBS 114405	0	0	1	4	2	2	1	10
Coccidioides immitis RS	0	1	5	4	7	3	1	21
Coccidioides posadasii C735 delta SOWgp	0	1	6	3	5	3	1	19
Coccodinium bartschii CBS 121709 v1.0	1	2	4	9	5	2	1	24
Cyphellophora europaea CBS 101466	0	0	4	5	2	2	0	13
Endocarpon pusillum Z07020	2	2	3	4	16	3	2	32
Eurotiomycetes sp. JF 03-3F Goopy v1.0	0	0	4	8	3	2	0	17
Eurotiomycetes sp. JF 03-4F Slimy v1.0	0	0	4	8	3	2	0	17
Eurotium rubrum v1.0	2	0	4	8	12	2	1	29

Genome	DMAT	HYBRID	NRPS	NRPS-Like	PKS	PKS-Like	TC	Total
Exophiala alcalophila ATCC 48519 v1.0	0	0	3	3	3	2	0	11
Exophiala alcalophila J33 v1.0	0	0	3	3	3	2	0	11
Exophiala aquamarina CBS 119918	0	0	4	8	5	5	1	23
Exophiala dermatitidis UT8656	0	0	4	3	2	3	0	12
Exophiala mesophila CBS40295	0	0	2	5	1	2	0	10
Exophiala oligosperma CBS72588	0	0	4	5	2	3	0	14
Exophiala sideris CBS121828	0	0	2	3	2	2	0	9
Exophiala spinifera CBS89968	0	0	3	8	3	2	0	16
Exophiala xenobiotica CBS118157	0	0	2	4	5	3	0	14
Fonsecaea monophora CBS 269.37	0	0	4	7	3	2	0	16
Fonsecaea multimorphosa CBS 102226	0	0	2	8	4	2	0	16
Fonsecaea nubica CBS 269.64	0	0	3	7	3	2	0	15
Fonsecaea pedrosoi CBS 271.37	0	0	3	8	3	2	0	16
Gymnascella aurantiaca v1.0	2	0	6	2	8	2	2	22
Gymnascella citrina v1.1	2	2	7	1	5	2	1	20
Histoplasma capsulatum NAm1	0	0	5	4	1	2	1	13
Microsporum canis CBS 113480	5	5	12	7	11	4	3	47
Monascus purpureus v1.0	0	2	7	5	4	5	0	23
Monascus ruber NRRL 1597 v1.0	0	3	7	5	10	3	0	28
Neosartorya fischeri NRRL 181	7	1	14	10	15	2	1	50
Paecilomyces niveus CO7 v1.0	1	7	7	13	19	2	1	50
Paecilomyces variotii CBS 101075 v1.0	0	3	5	8	6	2	3	27
Paecilomyces variotii CBS144490 HYG1 v1.0	0	3	7	7	6	2	3	28
Paracoccidioides brasiliensis Pb03	0	0	4	5	3	2	1	15
Paracoccidioides brasiliensis Pb18	0	0	3	6	3	2	1	15
Penicillium antarcticum IBT 31811	2	1	13	8	18	2	2	46
Penicillium atramentosum RS17 v1.0	1	3	10	14	13	2	3	46
Penicillium bilaiae ATCC 20851 v1.0	2	3	8	23	18	2	2	58
Penicillium brevicompactum 1011305 v2.0	2	1	13	16	22	2	4	60
Penicillium brevicompactum AgRF18 v1.0	2	1	15	15	24	2	3	62
Penicillium canescens ATCC 10419 v1.0	2	2	14	10	26	3	5	62
Penicillium chrysogenum Wisconsin 54-1255	1	2	7	15	18	3	3	49
Penicillium chrysogenum v1.0	0	2	9	14	18	3	4	50
Penicillium coprophilum IBT 31321	5	2	12	13	20	2	1	55
Penicillium decumbens IBT 11843	1	2	4	5	8	1	1	22
Penicillium digitatum PHI26	1	3	10	9	10	2	3	38
Penicillium digitatum Pd1	1	2	11	8	9	3	3	37

Genome	DMAT	HYBRID	NRPS	NRPS-Like	PKS	PKS-Like	TC	Total
Penicillium expansum ATCC 24692 v1.0	4	6	15	10	25	4	5	69
Penicillium expansum d1	3	6	16	10	21	3	3	62
Penicillium fellutanum ATCC 48694 v1.0	1	1	7	9	12	2	1	33
Penicillium flavigenum IBT 14082	4	2	11	15	18	3	4	57
Penicillium glabrum DAOM 239074 v1.0	2	3	5	22	21	1	1	55
Penicillium griseofulvum PG3	3	3	10	16	21	4	1	58
Penicillium italicum PHI-1	0	3	7	7	19	3	4	43
Penicillium janthinellum ATCC 10455 v1.0	2	1	9	10	14	3	4	43
Penicillium lanosocoeruleum ATCC 48919 v1.0	3	4	12	13	21	2	6	61
Penicillium nalgiovense FM193	2	1	9	16	12	5	1	46
Penicillium nordicum DAOMC 185683	3	1	19	12	20	3	2	60
Penicillium oxalicum 114-2	3	4	11	13	5	2	2	40
Penicillium polonicum IBT 4502	4	6	17	17	19	3	2	68
Penicillium raistrickii ATCC 10490 v1.0	3	5	13	16	23	3	4	67
Penicillium solitum IBT 29525	2	3	12	13	21	4	4	59
Penicillium solitum IBT 30036 v1.0	2	4	11	12	20	2	5	56
Penicillium sp. NC0857 v1.0	2	0	9	10	14	4	1	40
Penicillium steckii IBT 24891	4	3	10	13	11	2	0	43
Penicillium subrubescens FBCC1632 / CBS132785	3	1	11	12	21	3	0	51
Penicillium swiecickii 182 6C1 v1.0	5	3	14	15	32	3	5	77
Penicillium thymicola DAOMC 180753 v1.0	2	3	18	17	23	2	5	70
Penicillium vulpinum IBT 29486	3	5	12	11	29	2	5	67
Phaeomoniella chlamydospora UCRPC4	0	0	4	6	8	2	0	20
Phaeomoniellales sp. NC0930 v1.0	0	0	10	11	5	3	0	29
Phialophora attae CBS 131958	0	0	1	4	3	2	0	10
Talaromyces aculeatus ATCC 10409 v1.0	0	2	11	17	26	5	1	62
Talaromyces borbonicus CBS 141340	2	3	8	8	16	2	2	41
Talaromyces marneffei ATCC 18224	1	2	8	13	18	5	2	49
Talaromyces proteolyticus PMI_201 v1.0	4	2	8	13	40	3	1	71
Talaromyces stipitatus ATCC 10500	1	3	6	15	29	6	4	64
Thermoascus aurantiacus ATCC26904 v2.0	0	1	3	3	3	2	0	12
Thermoascus aurantiacus v1.0	0	1	3	3	3	2	0	12
Thermomyces lanuginosus SSBP	1	1	3	3	4	2	0	14
Trichophyton rubrum CBS 118892	1	3	13	2	8	2	2	31
Trichophyton verrucosum HKI 0517	3	3	12	5	7	2	2	34
Uncinocarpus reesii UAMH 1704	3	2	4	2	4	2	0	17
**MUCOROMYCOTINA**								

Genome	DMAT	HYBRID	NRPS	NRPS-Like	PKS	PKS-Like	TC	Total
Absidia caerulea NRRL1315 v1.0	0	0	1	1	1	1	0	4
Absidia padenii NRRL 2977 v1.0	0	0	1	1	1	1	0	4
Absidia repens NRRL 1336 v1.0	0	0	1	1	1	1	0	4
Amylomyces rouxii NRRL 5866 v1.0	0	0	1	7	2	1	0	11
Backusella circina FSU 941 v1.0	0	0	1	5	2	1	0	9
Benjaminiella poitrasii RSA 903 v1.0	0	0	1	5	2	1	0	9
Blakeslea trispora F921 v1.0	0	0	1	3	1	1	0	6
Blakeslea trispora F986 v1.0	0	0	1	3	1	1	0	6
Blakeslea trispora NRRL 2456 v1.0	0	0	1	3	1	1	0	6
Chaetocladium brefeldii NRRL 2343 v1.0	0	0	1	2	0	1	0	4
Choanephora cucurbitarum NRRL2744 (50) v1.0	0	0	1	5	1	1	0	8
Circinella umbellata NRRL1351 v1.0	0	0	0	4	1	1	0	6
Cokeromyces recurvatus NRRL 2243 v1.0	0	0	1	2	2	1	0	6
Cunninghamella echinulata NRRL 1382 v1.0	0	0	1	0	1	1	0	3
Dichotomocladium elegans RSA 919-v1.0	0	0	0	1	1	1	0	3
Dichotomocladium robustum RSA 2353+ v1.0	0	0	0	4	1	1	0	6
Ellisomyces anomalus CBS 243.57 v2.0	0	0	1	4	2	1	0	8
Endogone sp FLAS 59071	0	0	0	2	1	1	0	4
Fennellomyces linderi CBS 158.54 v1.0	0	0	0	5	1	1	0	7
Fennellomyces sp. T-0311 v1.0	0	0	0	5	1	1	0	7
Gilbertella persicaria var. persicaria CBS 190.32-T v1.0	0	0	1	3	1	1	0	6
Gongronella butleri v1.0	0	0	1	2	1	1	0	5
Halteromyces radiatus CBS 162.75 v1.0	0	0	1	1	1	1	0	4
Helicostylum pulchrum RSA 2064 v1.0	0	0	1	4	1	1	0	7
Hesseltinella vesiculosa NRRL3301 v2.0	0	0	1	2	1	1	0	5
Jimgerdemannia flammicorona AD002	0	0	0	2	0	1	0	3
Jimgerdemannia flammicorona GMNB39	0	0	0	1	1	1	0	3
Jimgerdemannia lactiflua OSC166217	0	0	0	3	0	1	0	4
Lentamyces parricidus NRRL2409 v1.0	0	0	0	6	1	1	0	8
Lentamyces zychae NRRL 2806 v1.0	0	0	0	4	1	1	0	6
Lichtheimia corymbifera JMRC:FSU:9682	0	0	0	5	1	1	0	7
Lichtheimia hyalospora v1.0	0	0	0	6	1	1	0	8
Mucor cordense RSA 1222 v1.0	0	0	1	7	2	1	0	11
Mucor endophyticus	0	0	1	2	1	1	0	5
Mucor fuscus	0	0	1	0	1	1	0	3
Mucor heterogamus NRRL 1489 v1.0	0	0	2	3	1	1	0	7
Mucor lanceolatus	0	0	1	0	1	1	0	3

Genome	DMAT	HYBRID	NRPS	NRPS-Like	PKS	PKS-Like	TC	Total
Mucor lusitanicus (circinelloides) MU402 v1.0	0	0	1	4	2	1	0	8
Mucor lusitanicus CBS277.49 v2.0	0	0	1	4	2	1	0	8
Mucor mucedo NRRL 3635 v1.0	0	0	1	4	1	1	0	7
Mucor racemosus	0	0	1	2	2	1	0	6
Mycotypha africana NRRL 2978 v1.0	0	0	1	1	2	1	0	5
Mycotypha indica IMI 211999 v1.0	0	0	1	2	2	1	0	6
Parasitella parasitica v1.0	0	0	1	2	2	1	0	6
Phascolomyces articulosus v1.0	0	0	0	7	1	1	0	9
Phycomyces blakesleeanus L51 v1.0	0	0	0	2	1	1	0	4
Phycomyces blakesleeanus NRRL1555 v2.0	0	0	1	2	1	1	0	5
Phycomyces blakesleeanus UBC1 v1.0	0	0	1	1	1	1	0	4
Phycomyces blakesleeanus UBC21 v2.0	0	0	1	3	1	1	0	6
Phycomyces nitens S607 v1.0	0	0	1	2	1	1	0	5
Phycomyces nitens S608 v1.0	0	0	1	2	1	1	0	5
Phycomyces nitens S609 v1.0	0	0	1	2	1	1	0	5
Pilaira anomala RSA1997 v1.0	0	0	1	3	1	1	0	6
Pilobolus umbonatus NRRL 6349 v1.0	0	0	1	0	1	1	0	3
Radiomyces spectabilis NRRL 2753 v1.0	0	0	0	3	1	1	0	5
Rhizopus delemar 99-880 from Broad	0	0	1	6	1	1	0	9
Rhizopus microsporus ATCC11559 v1.0	0	0	1	3	1	1	0	6
Rhizopus microsporus var. chinensis CCTCC M201021	0	0	2	6	1	2	0	11
Rhizopus microsporus var. microsporus ATCC52813 v1.0	0	0	1	3	1	1	0	6
Rhizopus microsporus var. microsporus ATCC52814 v1.0	0	0	1	3	1	1	0	6
Saksenaea vasiformis B4078	0	0	1	1	1	0	0	3
Spinellus fusiger NRRL 22323 v1.0	0	0	0	6	1	1	0	8
Sporodiniella umbellata MES 1446 v1.0	0	0	1	6	1	1	0	9
Syncephalastrum racemosum NRRL 2496 v1.0	0	0	0	7	1	1	0	9
Thamnidium elegans v1.0	0	0	1	4	1	1	0	7
Umbelopsis isabellina AD026 v1.0	0	0	1	5	1	2	0	9
Umbelopsis ramanniana AG # v1.0	0	0	1	4	0	1	0	6
Umbelopsis sp. PMI_123 v1.0	0	0	1	4	1	1	0	7
Umbelopsis sp. nov. AD052 v1.0	0	0	1	3	1	1	0	6
Zychaea mexicana RSA 1403 v1.0	0	0	0	3	1	1	0	5
